# Reaction of Fe_aq_^II^ with Peroxymonosulfate and Peroxydisulfate in the Presence of Bicarbonate: Formation of Fe_aq_^IV^ and Carbonate Radical Anions

**DOI:** 10.1021/acs.est.3c00182

**Published:** 2023-04-13

**Authors:** Aswin Kottapurath Vijay, Vered Marks, Amir Mizrahi, Yinghao Wen, Xingmao Ma, Virender K. Sharma, Dan Meyerstein

**Affiliations:** † Department of Chemical Sciences and The Radical Research Center, Ariel University, Ariel 40700, Israel; ‡ Chemistry Department, Ben-Gurion University, Beer-Sheva 8410501, Israel; § Department of Chemical Sciences, Ariel University, Ariel 40700, Israel; ∥ Chemistry Department, Negev Nuclear Research Centre, Beer-Sheva 84190, Israel; ⊥ Department of Civil and Environmental Engineering, Texas A&M University, College Station, Texas 77843, United States; # Program for the Environment and Sustainability, Department of Environmental and Occupational Health, Texas A&M University, College Station, Texas 77843, United States

**Keywords:** advanced oxidation processes, carbonate radical anion, fenton-like reactions, ferryl ion, persulfate radical anion

## Abstract

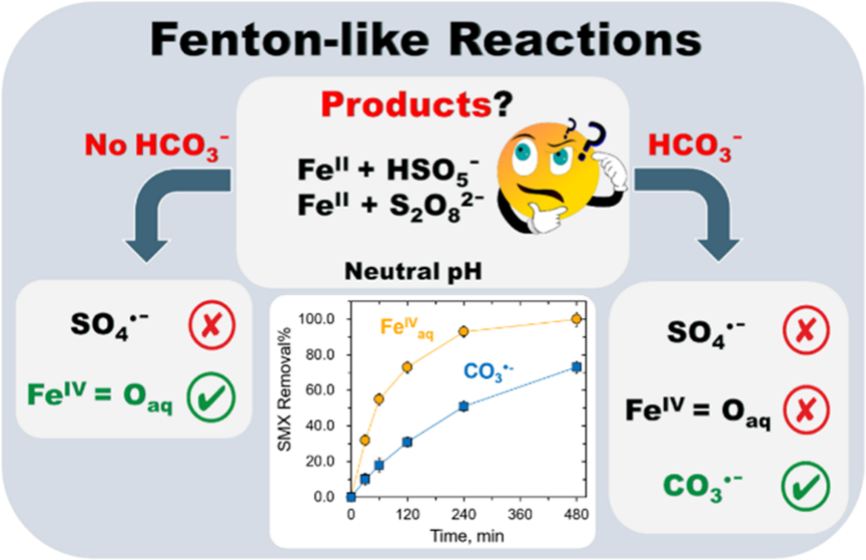

Many advanced oxidation processes (AOPs) use Fenton-like reactions to degrade organic pollutants by activating peroxymonosulfate (HSO_5_^–^, PMS) or peroxydisulfate (S_2_O_8_^2–^, PDS) with Fe(H_2_O)_6_^2+^ (Fe_aq_^II^). This paper presents results on the kinetics and mechanisms of reactions between Fe_aq_^II^ and PMS or PDS in the absence and presence of bicarbonate (HCO_3_^–^) at different pH. In the absence of HCO_3_^–^, Fe_aq_^IV^, rather than the commonly assumed SO_4_^•–^, is the dominant oxidizing species. Multianalytical methods verified the selective conversion of dimethyl sulfoxide (DMSO) and phenyl methyl sulfoxide (PMSO) to dimethyl sulfone (DMSO_2_) and phenyl methyl sulfone (PMSO_2_), respectively, confirming the generation of Fe_aq_^IV^ by the Fe_aq_^II^-PMS/PDS systems without HCO_3_^–^. Significantly, in the presence of environmentally relevant concentrations of HCO_3_^–^, a carbonate radical anion (CO_3_^•–^) becomes the dominant reactive species as confirmed by the electron paramagnetic resonance (EPR) analysis. The new findings suggest that the mechanisms of the persulfate-based Fenton-like reactions in natural environments might differ remarkably from those obtained in ideal conditions. Using sulfonamide antibiotics (sulfamethoxazole (SMX) and sulfadimethoxine (SDM)) as model contaminants, our study further demonstrated the different reactivities of Fe_aq_^IV^ and CO_3_^•–^ in the Fe_aq_^II^-PMS/PDS systems. The results shed significant light on advancing the persulfate-based AOPs to oxidize pollutants in natural water.

## Abstract 2

Iron(II) activation of peroxymonosulfate and peroxydisulfate generates Fe_aq_^IV^ and CO_3_^•−^ in the absence and presence of HCO_3_^−^, respectively, at neutral pH, resulting in different mechanisms and efficiencies for pollutant oxidation.

## Introduction

The increasing diversity and concentrations of organic contaminants in the environment have become a growing concern in past few decades.^1,2^ Many of these contaminants are recalcitrant and persistent, threatening the health of the ecosystem and human beings.^3^ Advanced oxidation processes (AOPs) are treatment technologies that utilize reactive species (e.g., hydroxyl radicals (OH^•^) and sulfate radical anions (SO_4_^•–^)) to break down organic contaminants in water.^4−6^ The redox potentials of OH^•^ and SO_4_^•–^ are in the ranges of +1.9 to 2.7 V_NHE_ and +2.4 (*E*^0^ (SO_4_^•–^/SO_4_^2–^ V_NHE_)), respectively, and can effectively oxidize many contaminants in water.^7−9^ The SO_4_^•–^-based AOPs have attracted greater attention in recent years because of its longer lifetime (30–40 μs) compared to that of OH^•^ (<1 μs).^10,11^ In addition, SO_4_^•–^ can be applied over a wider pH range and is more selective and has lower reactivity than OH^•^ toward interfering natural organic matter in water.^12,13^ SO_4_^•–^ are usually produced by activating peroxymonosulfate (PMS, HSO_5_^–^) or peroxydisulfate (PDS, S_2_O_8_^2–^) using ultraviolet or visible-light irradiation, carbonaceous materials, and transition metals.^6,14−20^ Among metal activators, iron(II) in water (Fe(H_2_O)_6_^2+^) is attractive because it is environmentally friendly and a number of studies have already demonstrated its efficacy in activating PMS and PDS.^21−25^ It was also shown that the Fenton reaction always proceeds via inner-sphere complexation due to thermodynamic reasons.^26−28^ It is generally assumed that the reactive-oxidizing species in these systems are formed via the following reactions (reactions 1 and 2).^29−34^
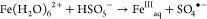
1
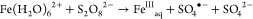
2where Fe_aq_^III^ is used as a general term to represent all Fe^III^ species in water, which changes with the pH and Fe^III^ concentration. In the last few years, however, studies are suggesting that the active product of reactions 1 and 2 in the pH range 3.0–9.0 is Fe_aq_^IV^, instead of SO_4_^•–^.^22,23^ A more recent study on the degradation kinetics of organic contaminants using the Fe(II)/PMS processes suggested that both Fe_aq_^IV^ and SO_4_^•–^ are involved in oxidizing contaminants.^35^ The discrepancies in the literature on the reactive species in the Fe(II)/PMS system prompted us to revisit the involved reactive species in the reactions of Fe(II) with PMS and PDS in neutral pH.

Notably, investigations on Fe(II)/PMS and Fe(II)/PDS systems in the literature rarely considered the possible effect of ubiquitous bicarbonate ion (HCO_3_^–^) at environmentally relevant concentrations as the solubility of CaCO_3_ at pH 7.0 is 3.0 mM. While HCO_3_^–^/CO_3_^2–^ is generally considered only as a buffer or a proton transfer agent, recent results suggest that HCO_3_^–^/CO_3_^2–^ are involved in a variety of important catalytic oxidation processes.^36−38^ Thus, studying the role of HCO_3_^–^/CO_3_^2–^ on reactions 1 and 2 is thus important. In particular, a recent study^39^ showed that the Fenton reaction (i.e., Fe(H_2_O)_6_^2+^ + H_2_O_2_) in the presence of bicarbonate yields a substantial amount of carbonate radical anions, CO_3_^•–^, rather than OH^•^ or Fe^IV^=O_aq_^2+^ (Fe_aq_^IV^) as commonly assumed. The same conclusion was obtained for the Fenton-like reaction in the presence of citrate.^40^ Significantly, the relative redox potentials of the CO_3_^•–^/CO_3_^2–^ and the (OH^•^ + H^+^)/H_2_O couples also suggest the formation of CO_3_^•–^ and not OH^•^ in the presence of HCO_3_^–^. Thus, the Fenton^39^ and Fenton-like reactions^40,41^ in the presence of HCO_3_^–^ may yield CO_3_^•–^ anion radicals via reactions between OH^•^ and HCO_3_^–^.^42^

The redox potential of the CO_3_^•–^/CO_3_^2–^ couple is only 1.57 V vs NHE,^43,44^ much lower than that of SO_4_^•–^ or OH^•^ even though the redox potential for the (CO_3_^•–^ + H^+^)/HCO_3_^–^ couple may be somewhat higher due to reaction 3 but remains lower than that of SO_4_^•–^ or OH^•^.
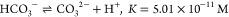
3Consequently, the formation of CO_3_^•–^ in the AOPs may have major ramifications because it is a weaker oxidant than SO_4_^•–^ or OH^•^. However, its lifetime is orders of magnitude longer than that of OH^•^.^45^ Furthermore, CO_3_^•–^ is considerably more selective^46^ and reacts via the inner-sphere mechanism in most systems.^43,47^ The HCO_3_^–^ present in the Fe(II)/PMS or Fe(II)/PDS systems may thus decrease the effectiveness of the system to oxidize pollutants in water.

The aims of the present study were to (i) demonstrate unequivocally the formation of Fe_aq_^IV^ in the reactions of Fe(II) and PMS or PDS in the absence of interfering chemicals, (ii) verify the generation of carbonate radical anions in the presence of bicarbonate in neutral solutions by investigating the kinetics and mechanisms of reactions of Fe_aq_^II^ with PMS/PDS under different conditions, and (iii) assess the implications of the newly confirmed mechanisms for the degradation of environmental pollutants with sulfonamides (sulfamethoxazole (SMX) and sulfadimethoxine (SDM)) as model pollutants.

## Experimental Methods

### Materials

All chemicals were of analytical grades and were used without further purification. Iron(II) perchlorate, potassium peroxymonosulfate, potassium peroxydisulfate, sodium bicarbonate, NaOH, and perchloric acid were acquired from Sigma-Aldrich (Rehovot, Israel). 2-(*N*-morpholino)ethanesulfonic acid (MES) was obtained from Chem-Impex Int’l Inc. Dimethyl sulfoxide was purchased from TCI. Deuterium oxide (D_2_O) was bought from Tzamal D-Chem Laboratories Ltd. Sulfamethoxazole (SMX, 98%) and phenyl methyl sulfoxide (PMSO, >98.0%) were purchased from Thermo Fisher Scientific (Waltham). Sulfadimethoxine (SDM, >98.0%) was acquired from TCI America (Portland). Waters Oasis HLB cartridges (WAT106202, 6 cc/200 mg) were obtained from Waters (Milford).

### Kinetics Study

Most of the experiments were conducted in a near-neutral pH by using a 0.60 mM 2-(*N*-morpholino)ethanesulfonic acid (MES) buffer solution, a non-coordinating tertiary-amine buffer with a p*K*_a_ of 6.06. The pH was adjusted to 7.40 ± 0.05 using NaOH. The pH measurements were made using a Schott Instrument Lab 850 pH meter. The kinetic studies by varying the pH (using NaOH and HClO_4_) of the solutions were also carried out. Stock solutions of both PMS and PDS (2.0 mM) were prepared in water. Stock solutions of iron(II) perchlorate (5.0 mM) in buffered Milli-Q H_2_O (Millipore) were made. The exact amount of iron(II) perchlorate crystals was added to the argon-saturated buffered solution while argon purging was running to avoid any contact of iron(II) with oxygen. All of the solutions were purged with argon in glass syringes during the preparation and before carrying out kinetic studies.

The kinetic measurements were performed using a stopped-flow SX20 from Applied Photophysics Ltd., equipped with a xenon arc lamp light source of 150 W. The optical path length of the measuring cuvette was 2.0 mm. All measurements were carried out under an argon atmosphere at 25 ± 0.10 °C. The solutions were injected into a mixing chamber (1:1), and the resulting mixture (here, Fe^II^aq and PMS or PDS (with or without bicarbonate)) traveled to an optical cell, where the change in the absorbance with time was measured. Thus, the pH in the kinetic runs was always pH 7.40 ± 0.05. The concentrations mentioned in the study are those in the final solutions. Single-wavelength kinetics data were collected at 270 nm to determine the rates of reactions. The experiments were repeated at least five times to assess the reproducibility.

Several difficulties arose in the study of the effect of [HCO_3_^–^] on the reaction rate. At pH 7.40, CO_2_ is also present in the solution. Removing the O_2_ by bubbling with an inert gas also drives CO_2_ out of the solution and decreases the HCO_3_^–^ concentration considerably. To overcome this problem, the argon gas was passed through a gas washing bottle containing a solution of HCO_3_^–^ at the same concentration. This method and its effectiveness were previously reported.^39^

### Reactive Species Measurements

Different analytical approaches were applied to determine reactive species involved in the studied system. DMSO ((CH_3_)_2_SO) reacts with Fe^IV^=O_aq_ by oxygen atom transfer forming dimethyl sulfone, (CH_3_)_2_SO_2_,^48^ while OH^•^ generates methyl-sulfinic acid (CH_3_SOOH) and a mixture of methane and ethane (via methyl radicals).^49^ DMSO was added to the solutions, and the products formed by the oxidation of DMSO via the Fenton-like reactions were measured in the absence and presence of bicarbonate. The different products were identified by nuclear magnetic resonance spectroscopy (^1^H NMR) and gas chromatography (GC). Specifically, ^1^H NMR measurements were performed on a 400 MHz Bruker Avance spectrometer. All samples were dissolved in solutions of H_2_O (90%)/D_2_O (10%), and the NMR experiments were performed at 300 K. The GC determination of methane and ethane was performed using an Agilent 7890B GC System with FID and TCD detectors and a GS Gaspro column.

As the concentrations of the reaction products in the stopped-flow experiments are too low to measure by the NMR method, the reactions were performed at higher concentrations. In this set of experiments, DMSO (25 mM) was added to the iron(II) solutions at the end of the preparations and the syringe was closed. Concentrated sodium bicarbonate solutions were prepared and injected into diluted PMS or PDS solutions in MES, pH ∼6.1, to form solutions containing the desired concentrations. Then, the pH was set to the required pH of 7.40 by adding NaOH or HClO_4_ as required. All stock solutions were prepared fresh prior to each set of experiments. Phenyl methyl sulfoxide (PMSO) was also used to probe the formation of Fe_aq_^IV^ in each treatment. Under the same condition as listed above, 20.0 and 200.0 μM PMSO were added to each tube in the PMS and PDS system, respectively. The concentrations of PMSO and its oxidation product phenyl methyl sulfone (PMSO_2_) in each sample at time = 10, 30, 60, 90, and 120 min were determined using a high-performance liquid chromatography (HPLC) method.^50^

### Electron Paramagnetic Resonance Experiment

EPR measurements were conducted using a Bruker Elexsys E500 EPR equipped with a CoolEdge cryo system (Billerica). The instrument settings were 20.0 mW microwave power, 9.8 GHz microwave frequency, 100 kHz modulation frequency, 1.00 G modulation amplitude, 3515 G center field, 150 G sweep width, and 40.0 s sweep time. The mixture of ultrapure water and acetonitrile (1:1) was used as the solvent. 50.0 mM 5,5-dimethyl-1-pyrroline *N*-oxide (DMPO) was used as the spin-trapping agent for reactive radical species. 1.0 mL of the reaction solution was extracted and injected into a 2 mm quartz EPR tube using a syringe needle. The 2 mm quartz tube was then placed into a 4 mm quartz EPR tube and immediately inserted into the EPR.

### Degradation of Sulfonamides

The degradation efficiencies of SMX and SDM were determined in six different systems as listed in Table S1. To examine the effect of bicarbonate, the bicarbonate concentration was varied at 0, 0.05, 0.5, 5.0, and 20.0 mM in the PMS systems and 0, 0.5, 5.0, 50.0, and 200.0 mM in the PDS systems. The degradation experiments were conducted in 40 mL glass tubes with caps, which were covered with aluminum foil to avoid the interference of light. The initial pH of the reaction solution in each tube was adjusted to 7.0 ± 0.2 using 0.1 M NaOH and 0.1 M H_2_SO_4_. At each elapsed time point (*t* = 0, 30, 60, 120, 240, 480 min), 1.0 mL of the sample was extracted from each tube and immediately quenched by 0.2 mL of 0.5 M Na_2_S_2_O_3_. The concentration of SMX or SDM in each sample was measured using the high-performance liquid chromatography (HPLC) method. An instrument used was a Dionex UltiMate 3000 (Sunnyvale) and the column was a Restek C18 column (4.6 × 250 mm^2^, 5 μm).

## Results and Discussion

### Kinetics

The reactions of Fe_aq_^II^ with PMS or PDS at pH 7.4 were first investigated by monitoring the formation of Fe^III^ at 270 nm as a function of time. In this set of experiments, the concentration of HCO_3_^–^ was kept constant. Typical kinetic curves of the reactions under different concentrations of PMS and PDS are presented in Figures S1 and S2. The kinetic traces could be nicely fitted by exponential curves, suggesting that the rates are first order with respect to the concentration of Fe_aq_^II^. This was further confirmed by varying the concentrations of Fe_aq_^II^. The kinetic traces are given in Figures S3–S6. The observed first-order rate constants (*k*_obs_, s^–1^) did not change with the concentration of Fe_aq_^II^ (Figures S7 and S8), again supporting that the rates are first order with respect to [Fe_aq_^II^]. The distribution of different Fe(II) species in the presence of low (0.3 mM for PMS and 2.0 mM for PDS) and high (0.6 mM for PMS and 5.0 mM for PDS) concentrations of HCO_3_^–^ under neutral conditions was studied. The kinetics of the pH dependence (pH = 2.40–8.50) in the absence of bicarbonate were also conducted. Their kinetic traces are given in Figures S9 and S10. The observed rate constants (*k*_obs_, s^–1^) had no dependence on the solution pH (Figure S11). It should be noted that the precipitation of Fe(III) as indicated by the decrease of the observed light absorption was observed only after several minutes.

**Figure 1:**
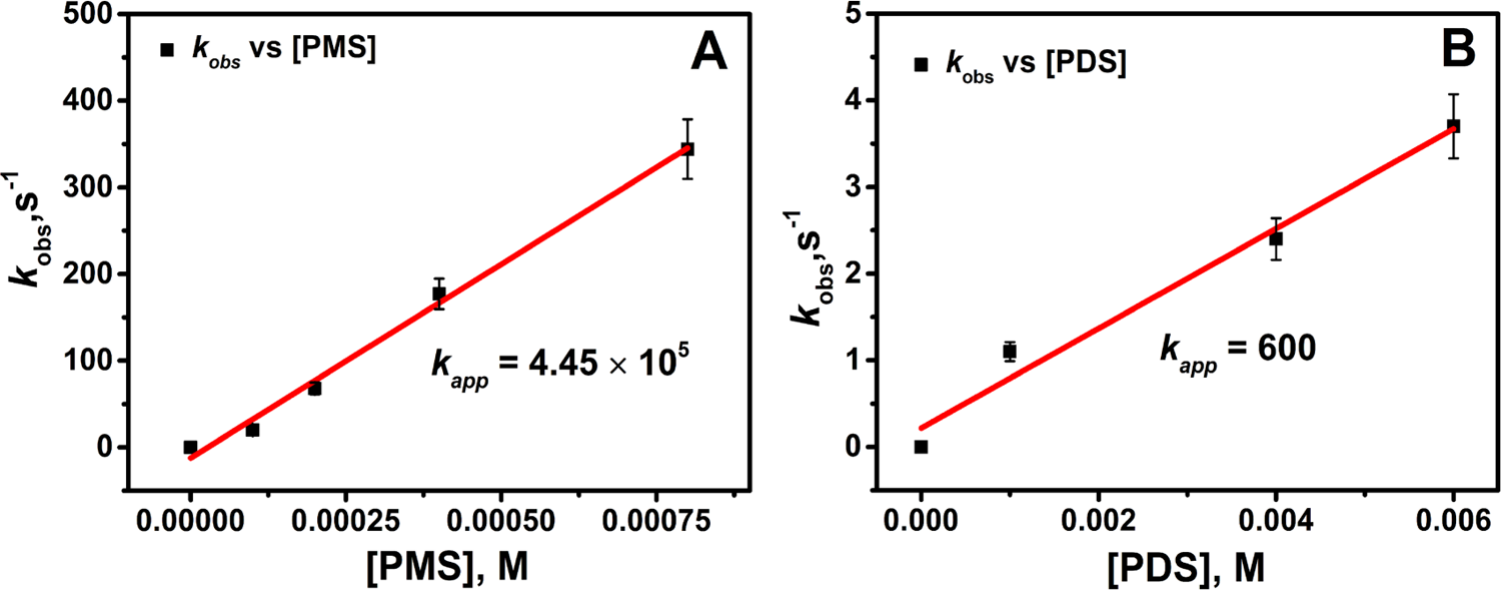
Dependence of *k*_obs_ at a constant concentration of HCO_3_^–^ on the concentration of peroxymonosulfate (PMS) and peroxydisulfate (PDS) at pH 7.4. (A) PMS (HSO_5_^–^). [Fe_aq_^II^] = 0.020 mM, [HCO_3_^–^] = 0.30 mM. (B) PDS (S_2_O_8_^2–^). [Fe_aq_^II^] = 0.10 mM, [HCO_3_^–^] = 4.0 mM. Percentage of error = ±10.

The variation of *k*_obs_ with the concentrations of PMS or PDS is presented in Figure 1. The linear dependence of *k*_obs_ on the concentrations of PMS and PDS indicates that the oxidation of Fe_aq_^II^ was due to the peroxides (i.e., PMS and PDS). Significantly, PMS reacts much faster than PDS with Fe_aq_^II^. Importantly, the potential precipitation of Fe(III) oxide can be ruled out in this study because the time required for the nucleation and formation of precipitates is much longer than the time scale of our experiments. The removal of Fe(III) through Fe(II)–Fe(III) also has a slower kinetics than the reaction of Fe(II) with PMS/PDS.

**Figure 2:**
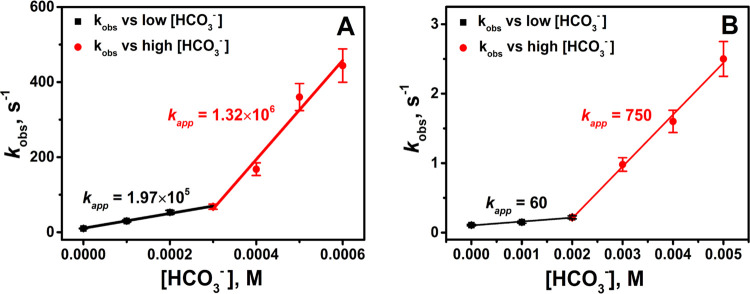
Dependence of *k*_obs_ on the concentration of HCO_3_^–^ for the reactions of Fe_aq_^II^ with PMS and PDS ^–^ at pH 7.4. (A) PMS, [Fe_aq_^II^] = 0.020 mM, [HSO_5_^–^] = 0.20 mM and (B) PDS, [Fe_aq_^II^] = 0.10 mM, [S_2_O_8_^2–^] = 1.0 mM. In both experiments, excess peroxymonosulfate (PMS) and peroxydisulfate (PDS) were used. Percentage of error = ±10.

Next, the kinetics of the reactions of Fe_aq_^II^ with PMS and PDS were studied at different concentrations of HCO_3_^–^. The results of *k*_obs_ at different concentrations of HCO_3_^–^ are shown in Figure 2A,B. Typical kinetic curves are presented in Figures S12 and S13. The addition of low concentrations of HCO_3_^–^ to the solutions increased the rate of reactions of Fe(H_2_O)_6_^2+^ with both PMS and PDS. The results are very similar to those obtained for the Fenton reaction^39^ that the rate constants depend linearly on HCO_3_^–^ but with two different slopes: a relatively low slope at a very low HCO_3_^–^ concentration (below 0.3 mM for PMS and below 2.0 mM for PDS, see Figure 2) and a considerably higher slope at higher values of HCO_3_^–^.

The rate law for the reaction of Fe_aq_^II^ and PMS/PDS in the presence of HCO_3_^–^ may be written as
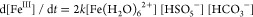
4
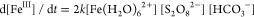
4′In eqs 4 and 4′, the coefficient 2 was derived from the observation that the oxidizing species formed in the system including OH^•^, SO_4_^•–^, Fe^IV^O_aq_, and CO_3_^•–^ oxidizes a second Fe_aq_^II^.

The two-stage dependence of *k*_obs_ on [HCO_3_^–^], shown in Figure 2, may be understood first by considering the different species of Fe_aq_^II^ present in the studied conditions. At low [HCO_3_^–^], the species of Fe_aq_^II^ is not complexed with HCO_3_^–^ (i.e., Fe(H_2_O)_6_^2+^). However, at higher concentrations of HCO_3_^–^, the following equilibria need to be considered (eqs 5–7).^51−55^ This suggests the complex formation of Fe_aq_^II^ with CO_3_^2–^ (i.e., Fe^II^(CO_3_)(H_2_O)_3_).^51^
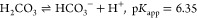
5(this is an apparent value as [H_2_CO_3_]_app_ = [H_2_CO_3_] + [CO_2_] is used).
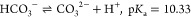
6


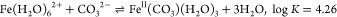
7

Three possible reaction mechanisms, I, II, and III, may be considered to describe the results presented in Figures 1 and 2 and the experimentally observed rate laws. Mechanism I at low concentrations of HCO_3_^–^ presumes that reactions 8, 8′, 9, and 9′ occur. Initially, the Fe^II^ forms a complex with PMS/PDS in the absence or in the presence of low bicarbonate (reactions 8 and 8′). The formed complexes then react with HCO_3_^–^ to generate the carbonate anion radicals (reactions 9 and 9′). The derived rate law for reactions 8, 8′, 9, and 9′ is consistent with the observed rate law (eqs 4 and 4′) (see Text S1). However, at higher concentrations of HCO_3_^–^, the complexation of Fe_aq_^II^ and bicarbonate is more dominant and is formed before reacting with PMS/PDS (eqs 10 and 10′). The direct Fe_aq_^II^–HCO_3_^–^ complexation and abundant PMS/PDS in the system facilitate the rapid production of reactive Fe_aq_^IV^ iron and carbonate anion species, leading to a steeper slope in Figure 2. The derived rate law is also consistent with the observed rate law (see Text S1).

#### Mechanism I


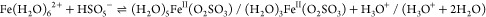
8
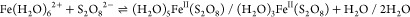
8′
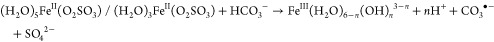
9
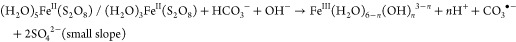
9′
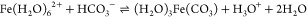
10
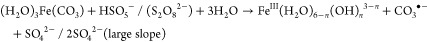
10′

While more complicated complexes could potentially form in the system that also results in a two-stage dependence on the bicarbonate concentration for the system, these mechanisms are unlikely in our system because both mechanisms require a second-order dependence on [HCO_3_^–^] that is not observed (Text S2 (eqs 11–14) and Text S3 (eqs 15–18), Supporting Information). It should be pointed out that mechanisms II and III might contribute at very high bicarbonate concentrations. This possibility is further discussed in the degradation of investigated sulfonamides by studied Fenton-like systems at neutral pH.

Overall, mechanism I fits the observed rate laws. The rate constants of reactions 9 and 9′ cannot be calculated because the equilibrium constants of reactions 8 and 8′ are not known. The rate constant of the reactions (H_2_O)_3_Fe(CO_3_) + HSO_5_^–^ and (H_2_O)_3_Fe(CO_3_) + S_2_O_8_^2–^ can be roughly calculated by dividing the larger slope in Figure 2A, 1.32 × 10^6^ M^–1^ s^–1^ by [HSO_5_^–^] = 0.0002 M, and that in Figure 2B, 750 dm^3^ mol^–1^ s^–1^ by [S_2_O_8_^2–^] = 0.001 M, respectively. Thus, one obtains *k*(Fe^II^(CO_3_)(H_2_O)_3_ + HSO_5_^–^) = 6.6 × 10^9^ M^–2^ s^–1^ and *k*(Fe^II^(CO_3_)(H_2_O)_3_ + S_2_O_8_^2–^) = 7.5 × 10^5^ M^–2^ s^–1^. It is important to note that a large error limit must be applied on these values mainly because some CO_2_ might have been lost from the solutions during bubbling. The value of *k*(Fe^II^(CO_3_)(H_2_O)_3_ + HSO_5_^–^) is higher by 1 order of magnitude than that of *k*(Fe^II^(CO_3_)(H_2_O)_3_ + H_2_O_2_) = 5.5 × 10^8^ M^–1^s^–1^^39^ and more than 5 orders of magnitude higher than those of *k*(Fe^II^(P_2_O_7_)_aq_ + H_2_O_2_)^56^ = 3500 dm^3^ mol^–1^ s^–1^ and *k*(Fe^II^(ATP)_aq_ + H_2_O_2_)^56^ = 1200 M^–1^ s^–1^ though both ATP and P_2_O_7_^4–^ clearly stabilize Fe_aq_^III^ better than carbonate.^56^

### Reactive Species in the Presence and Absence of Bicarbonate Ion

The above kinetic results clearly demonstrate that low concentrations of bicarbonate within the range typically observed in natural environments could affect the kinetics of the Fenton-like reactions in neutral solutions dramatically. To determine the nature of the oxidizing products formed in reactions 8 and 10′, (CH_3_)_2_SO was added to the reaction mixtures and ^1^H NMR spectra of the products of (CH_3_)_2_SO oxidation via the Fenton-like reaction were measured. Additionally, the gaseous products were also analyzed in the absence and presence of bicarbonate (3.0 mM).

**Figure 3:**
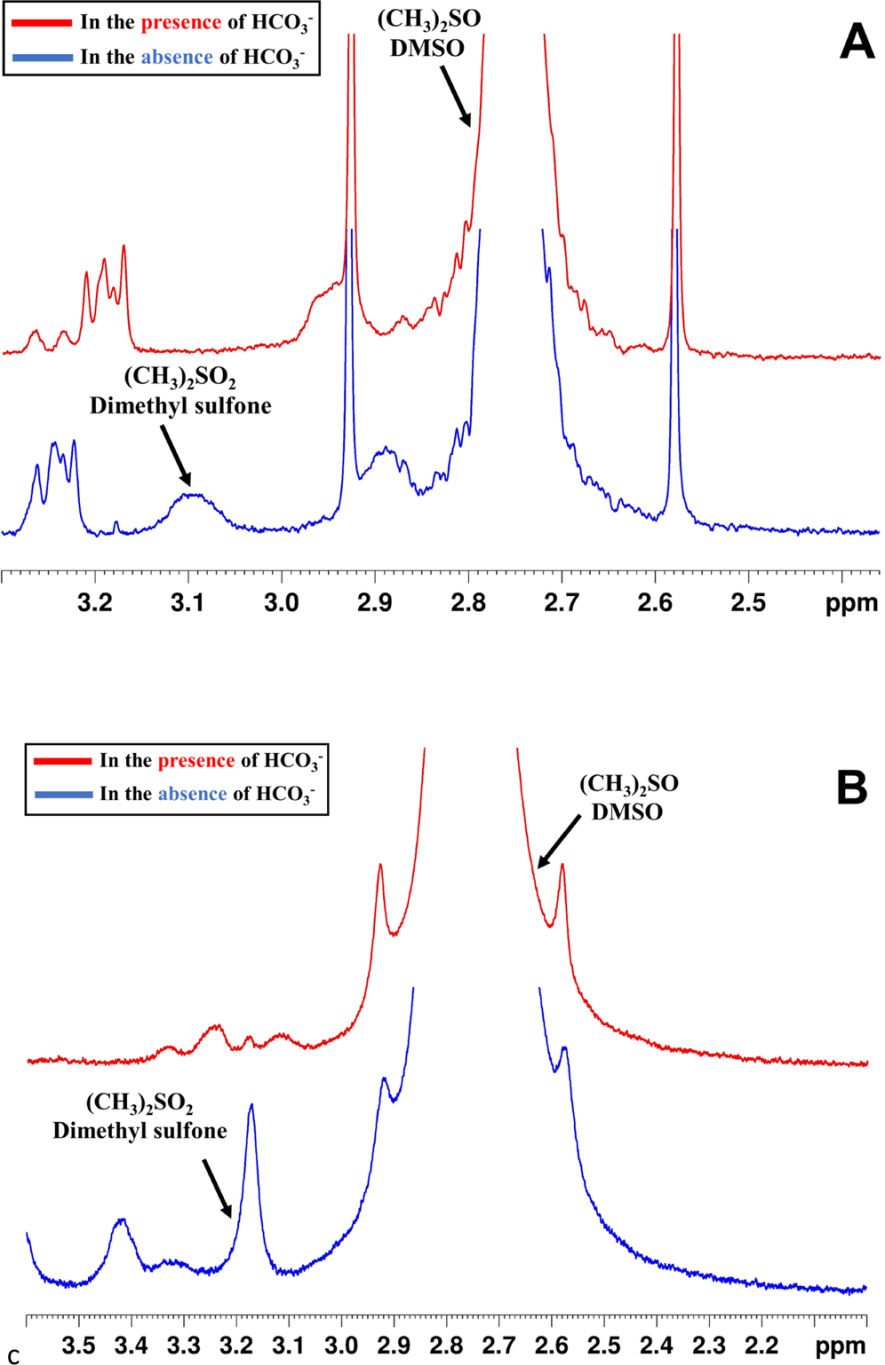
^1^H NMR spectra of the products of the Fenton-like reactions at neutral pH in MES buffer solution in H_2_O: (A) Fe^2+^ + HSO_5_^–^ in the absence and presence of 0.50 mM HCO_3_^–^, [Fe_aq_^II^] = 0.20 mM, [HSO_5_^–^] = 0.04 mM, and [(CH_3_)_2_SO] = 25.0 mM and (B) Fe^2+^ + S_2_O_8_^2–^ in the absence and presence of 5.0 mM HCO_3_^–^, [Fe_aq_^II^] = 2.0 mM, [S_2_O_8_^2–^] = 1.0 mM, and [(CH_3_)_2_SO] = 25.0 mM. The concentrations of bicarbonate chosen for these experiments are those that were shown, Figure 2, to have a major effect on the observed rate constants.

It is known that (CH_3_)_2_SO reacts with Fe^IV^=O_aq_ to form dimethyl sulfone, (CH_3_)_2_SO_2_,^48^ while reactions with OH^•^ and some other radicals generate methyl-sulfinic acid (CH_3_SOOH) and methyl radicals.^48,49^ The presence of bicarbonate, Figure 3, clearly affects the yield of (CH_3_)_2_SO_2_. Both in the presence of excess HSO_5_^–^/S_2_O_8_^2–^ (PMS/PDS) and excess Fe^2+^, the presence of a low concentration of bicarbonate inhibits the formation of (CH_3_)_2_SO_2_. CH_3_SOOH was also not observed as a product (see Figure 3A,B), which is analogous to the recent report on the reactions observed when H_2_O_2_ is used as the peroxide.^39^ The presence of 0.50 mM bicarbonate nearly eliminates the formation of (CH_3_)_2_SO_2_ in the case of HSO_5_^–^, whereas in S_2_O_8_^2–^, 5.0 mM bicarbonate is needed. Furthermore, the formation of (CH_3_)_2_SO_2_ as the final organic product proves that in the absence of bicarbonate, the Fenton-like reactions studied proceed via reactions 19 and 19′, which are in agreement with the previous results.^22,23,57−59^
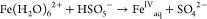
19
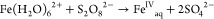
19′

This means that even in the absence of HCO_3_^–^, the Fenton-like reactions of Fe(H_2_O)_6_^2+^ + HSO_5_^–^/S_2_O_8_^2–^ do not form SO_4_^•–^ and/or OH^•^ as commonly assumed.^29−31^ To further illustrate its environmental significance, the effect of pH was conducted by varying the pH from acidic (2.40) to alkaline (8.50). The results of GC measurement of the gas products are presented in Figure S14, which showed that the sulfate anion radical (SO_4_^•–^) was formed in acidic pH and its yield decreased with the increase of pH. Therefore, the yield of methane and ethane also decreased with the increase in pH, Figure S14. The results could be attributed to the dominant role of Fe_aq_^IV^ in the absence of bicarbonate at neutral pH. To support the formation of CO_3_^•–^ in the Fenton-like reactions in the presence of bicarbonate and (CH_3_)_2_SO, the yields of CH_4_ and C_2_H_6_ under three different conditions were measured: (I) at pH 2.4 in the absence of bicarbonate; (II) at pH 7.4 in the absence of bicarbonate; and (III), at pH 7.4 in the presence of 3.0 mM bicarbonate. The results are presented in Figure S15. It is known that SO_4_^•–^ reacts with (CH_3_)_2_SO to form methyl radicals via reaction 20.^60^
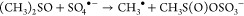
20However, also, other radicals react with (CH_3_)_2_SO to form methyl radicals.^60,61^ The formed methyl radicals (CH_3_^•^) (reaction 21) react to form ethane and methane via reactions 21–23.^46,62^
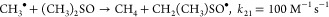
21


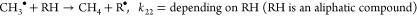
22


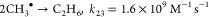
23

Ethane and methane are formed when the oxidizing species is a single-electron oxidizing agent, primary oxidizing radicals, such as SO_4_^•–^.^63^ The relative yields of ethane and methane depend on the steady-state concentrations of methyl radicals. The results at pH 2.4 for the PMS system clearly show that ethane is almost the only product. This suggests that at pH 2.4, SO_4_^•–^ is the major product of the reaction of Fe(H_2_O)_6_^2+^ + HSO_5_^–^. However, it was claimed that at pH 3.0, Fe_aq_^IV^ is the only product.^22,23,57−59^ Interestingly, in the PDS system, a considerable amount of methane was also formed. This is likely due to that *k*(Fe(H_2_O)_6_^2+^ + HSO_5_^–^) > *k*(Fe(H_2_O)_6_^2+^ + S_2_O_8_^2–^). At pH 7.4 in the HSO_5_^–^ system, no ethane was formed in the absence of HCO_3_^–^, and only traces of methane were observed. These results indicate that the product of reaction 19 is indeed Fe^IV^=O_aq_. At pH 7.4 in the PDS system in the absence of HCO_3_^–^, traces of ethane and some methane are observed. This supports the conclusion that the major product of reaction 19′ is Fe^IV^=O_aq_, though some Fe_aq_^III^ and SO_4_^•–^ are also formed. The fact that no CH_4_ and/or C_2_H_6_ were formed in the presence of bicarbonate in both systems, which further ruled out that SO_4_^•–^ radical anions were formed under these conditions.

**Figure 4:**
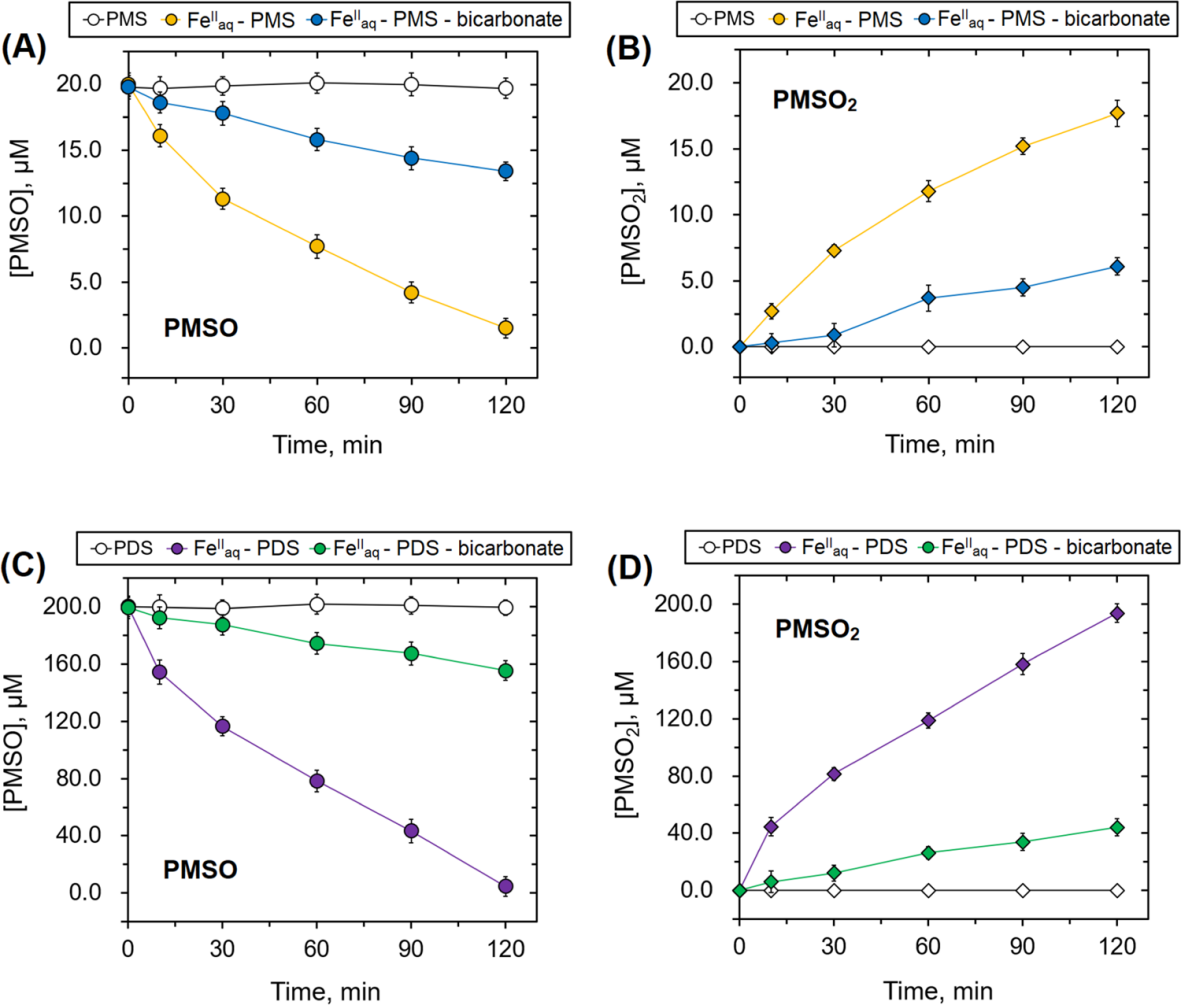
Changes in concentrations of PMSO and PMSO_2_ in different persulfate systems over time at initial pH = 7.0 (experimental conditions: PMS system: [PMS]_0_ = 0.04 mM; [Fe_aq_^II^]_0_ = 0.2 mM; [bicarbonate]_0_ = 0.5 mM; [PMSO]_0_ = 20.0 μM and PDS system: [PDS]_0_ = 1.0 mM; [Fe_aq_^II^]_0_ = 2.0 mM; [bicarbonate]_0_ = 5.0 mM; [PMSO]_0_ = 200.0 μM).

In order to further confirm the formation of high-valent iron species, PMSO was employed as the probing molecule, which can be selectively oxidized by Fe_aq_^IV^ or Fe_aq_^V^ to produce phenyl methyl sulfone (PMSO_2_).^64^ As shown in Figure 4, the concentration of PMSO remained unchanged with PMS or PDS alone, and PMSO_2_ was not generated. However, a rapid transformation from PMSO to PMSO_2_ was observed when Fe(II) was added, confirming the formation of Fe_aq_^IV^, similar to the results of using DMSO as the probe molecule (see Figure 3). The inhibitory effect of bicarbonate ion on this transformation could be attributed to the production of carbonate radical anions through mechanism **I** discussed earlier. Significantly, the formation of PMSO_2_ was not eliminated in the presence of 5.0 mM bicarbonate ion (Figure 4). This is somewhat different from the results presented in Figure 3 using DMSO as a probe molecule for the formation of Fe_aq_^IV^ in which no formation of DMSO_2_ was observed in 4.0 mM bicarbonate ion. In using PMSO, no formation of PMSO_2_ was seen only at a much higher concentration of bicarbonate ion (200 mM) (Figure S16). This may be related to differences in reactivity of (CO_3_)Fe_aq_^IV^ or Fe_aq_^IV^ with the probe molecules, DMSO and PMSO. This tentatively suggests that reaction 10′ is likely involved more complex reactions as shown below
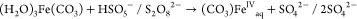
10″

Followed by
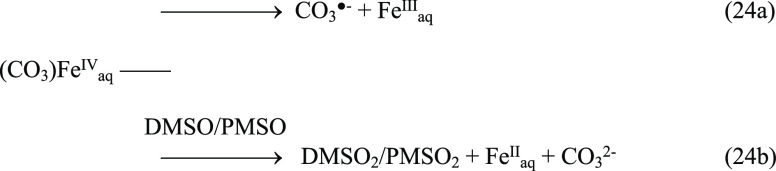
24

with the assumption that for DMSO, *k*_24a_ ≫ *k*_24b (DMSO)_ and for PMSO, *k*_24a_∼ *k*_24b (PMSO)_ and *k*_24b (PMSO)_ > *k*_24b (DMSO)_.

**Figure 5:**
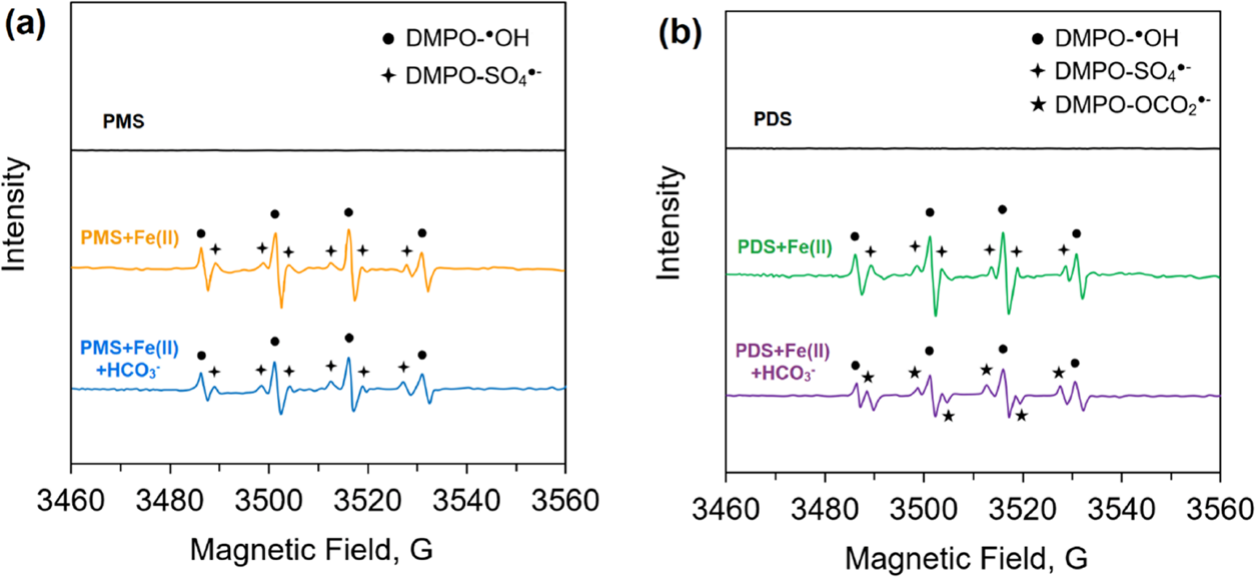
EPR spectra of (a) PMS and (b) PDS alone, with Fe(II) and with Fe(II) + HCO_3_^–^ (initial pH: 7.0; the PMS system: [PMS]_0_ = 0.04 mM, [Fe(II)]_0_ = 0 or 0.2 mM, [HCO_3_^–^]_0_ = 0 or 0.5 mM; the PDS system: [PDS]_0_ = 1.0 mM, [Fe(II)]_0_ = 0 or 2.0 mM, [HCO_3_^–^]_0_ = 0 or 5.0 mM).

In order to acquire direct evidence for the interactions between PMS/PDS, Fe(II), and HCO_3_^–^, EPR spectroscopy was employed to probe the signals of possible radical species. Without adding Fe(II), no signal was observed. In the presence of Fe(II), however, the signals of DMPO-^•^OH and DMPO–SO_4_^•–^ adducts were captured for both PMS and PDS (Figure 5a,b). Significantly, the signal of a new species was observed along with DMPO–^•^OH and DMPO–SO_4_^•–^ when HCO_3_^–^ was introduced into the PDS system, which could be attributed to the formation of DMPO–OCO_2_^•–^ adduct as a result of the binding between CO_3_^**•**–^ and DMPO.^65^ Several peaks of DMPO–SO_4_^•–^ and DMPO–OCO_2_^•–^ were overlapping with each other, suggesting that both SO_4_^•–^ and CO_3_^•–^ were present. Also, the nucleophilic substitution by hydroxide or water molecule can occur on the carbonate/sulfonate moieties in DMPO–OCO_2_^•–^/DMPO–SO_4_^•–^ via an exergonic process to produce DMPO–^•^OH.^65−67^ Overall, EPR measurement confirmed our hypothesis that CO_3_^**•**–^ is a major radical species in the Fe(II)–PMS/PDS system in the presence of HCO_3_^–^.

### Degradation of Sulfamethoxazole and Sulfadimethoxine

**Figure 6:**
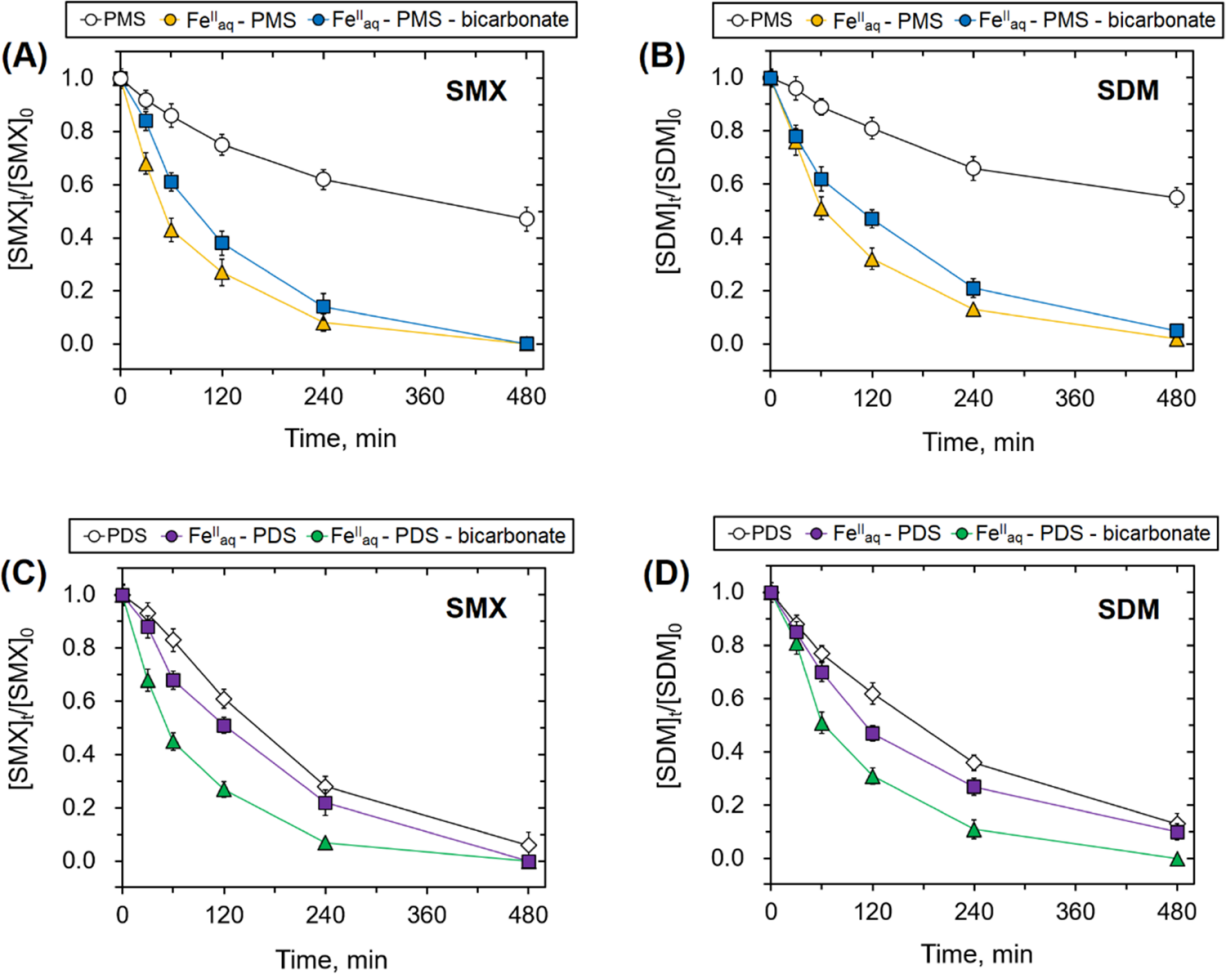
Degradation of sulfamethoxazole (SMX) and sulfadimethoxine (SDM) by (A, B) PMS and (C, D) PDS alone, in the presence of Fe_aq_^II^ and Fe_aq_^II^—bicarbonate ([SMX]_0_ = [SDM]_0_ = 5.0 μM; initial pH = 7.0. The PMS system: [PMS]_0_ = 0.04 mM; [Fe_aq_^II^]_0_ = 0.2 mM; [bicarbonate]_0_ = 0.5 mM. The PDS system: [PDS]_0_ = 1.0 mM; [Fe_aq_^II^]_0_ = 2.0 mM; [bicarbonate]_0_ = 5.0 mM).

Sulfonamides (SAs) have been extensively used as veterinary and human antibiotics. They can enter the human food chain and trigger the development of antibiotic resistance (AR).^68−70^ It is imperative to treat SAs before releasing them into the aquatic environment. The investigated systems herein were therefore investigated to degrade SAs, which contain an aniline ring and a heterocyclic N-containing aromatic ring (R) that are joined through a sulfonamide linkage (−NH–SO_2_^–^). The studied SMX and SDM are SAs with different R of five- and six-membered rings, respectively. The reaction of SMX with high-valent iron opens the five-membered ring, while no such ring opening happens in the case of SDM. Furthermore, there is no extrusion of SO_2_ in the case of SMX, whereas the loss of SO_2_ in the oxidized products was seen for SDM. These findings in the literature led us to select these sulfonamides, where the oxidized products in the reactions of high-valent iron, Fe(IV), with SMX and SDM could be examined. The degradation of such SAs by Fe_aq_^II^-PMS and Fe_aq_^II^-PDS systems in the absence and presence of bicarbonate ion may be extended to a wide range of SAs. The results obtained on the degradation efficiency at pH 7.0 are shown in Figure 6. The observed first-order rate constants (*k*_obs_) for the degradation of SMX and SDM are presented in Figure S17. The presence of Fe_aq_^II^ substantially enhanced the degradation efficiency of SMX and SDM by both persulfate systems (i.e., PMS and PDS). The *k*_obs_ for the degradation of SMX and SDM by PMS reached 1.0 × 10^–2^ and 0.8 × 10^–2^ min^–1^, respectively, in the presence of Fe_aq_^II^, which were 6-fold compared to PMS alone. In the case of PDS, the *k*_obs_ values for SMX and SDM in the presence of Fe_aq_^II^ were 1.1 × 10^–2^ and 0.9 × 10^–2^ min^–1^, respectively, about twice as high as PDS alone. The results suggest that the Fe_aq_^IV^ formed reacts with SMX and SDM with high reactivity to increase their oxidation.

**Figure 7:**
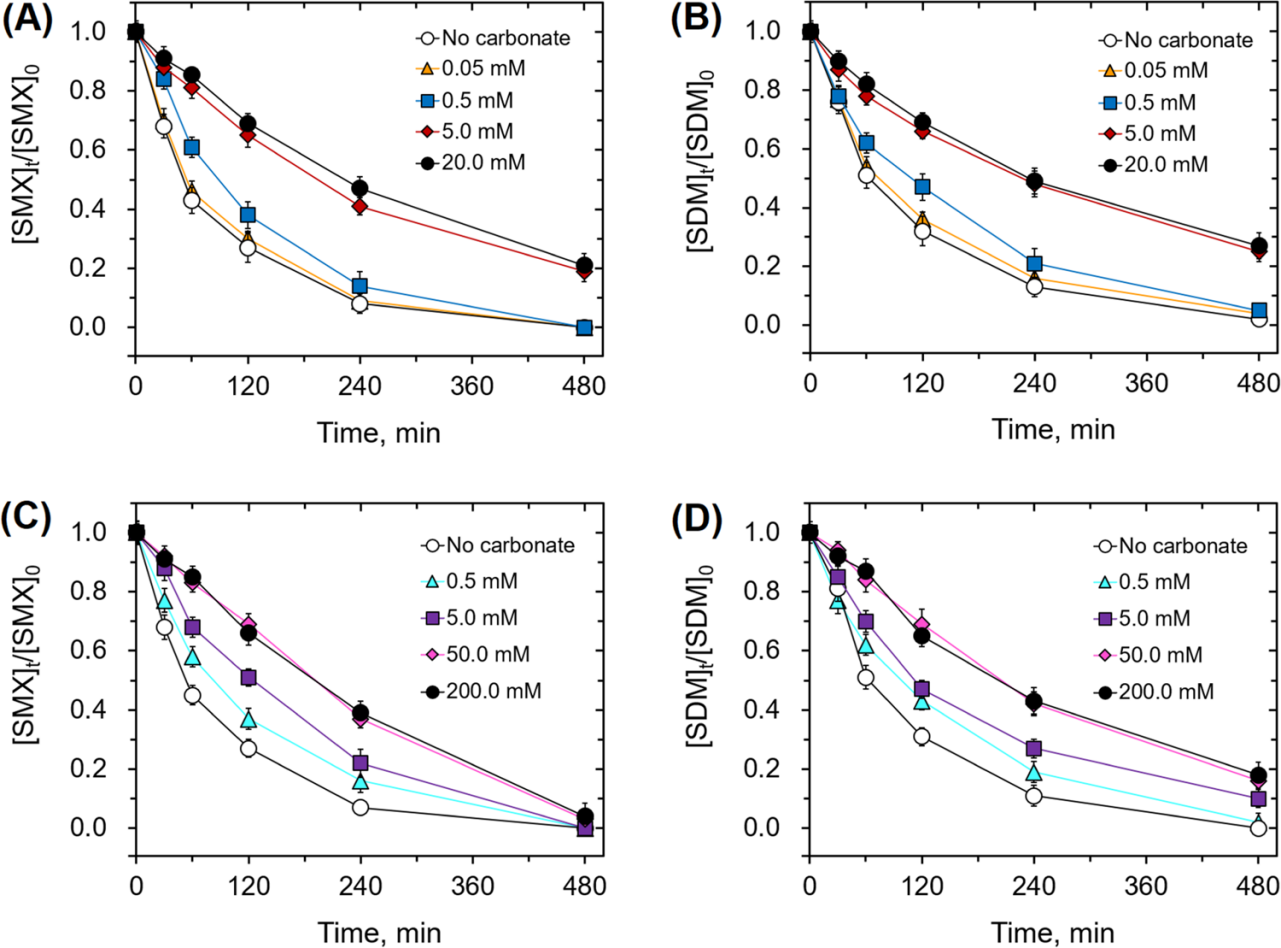
Degradation of SMX and SDM in the presence of varying carbonate concentrations in the (A, B) PMS and (C, D) PDS systems ([SMX]_0_ = [SDM]_0_ = 5.0 μM; initial pH = 7.0. The PMS system: [PMS]_0_ = 0.04 mM; [Fe_aq_^II^]_0_ = 0.2 mM; [bicarbonate]_0_ = 0, 0.05, 0.5, 5.0, and 20.0 mM. The PDS system: [PDS]_0_ = 1.0 mM; [Fe_aq_^II^]_0_ = 2.0 mM; [bicarbonate]_0_ = 0, 0.5, 5.0, 50.0, and 200.0 mM).

The bicarbonate ion markedly hinders the degradation efficiency of SMX and SDM by the Fe_aq_^II^-persulfate systems (Figure 7). As the bicarbonate concentration increased to above 0.5 and 5.0 mM for the PMS and PDS systems, the degradation efficiencies of SMX and SDM are further impeded. For example, with 5.0 mM bicarbonate, the *k*_obs_ for the degradation of SMX and SDM in the PMS system was decreased by 66.0 and 65.1%, respectively, compared to that without bicarbonate. On the other hand, 50.0 mM bicarbonate led to a 58.7 and 58.1% reduction in the *k*_obs_ for the degradation of SMX and SDM in the PDS system. However, as the bicarbonate concentration further increased from 5.0 to 20.0 mM in the PMS system and from 50.0 to 200.0 mM in the PDS system, further decreases in the degradation efficiency were very minor. The hindering effect of bicarbonate is likely due to the formation of the less reactive carbonate radical anion as confirmed above. This is consistent with kinetics analysis at a high concentration of HCO_3_^–^. This is supported by the complete inhibition of the transformation of PMSO to PMSO_2_ for PMS and PDS systems in the presence of 20.0 and 200.0 mM bicarbonate ion, respectively (see Figure S16).

## Environmental Significance

The results reported in this study are of major importance to understanding the mechanisms involved in many advanced oxidation processes. First, it is shown that the reactions of Fe_aq_^II^ with PMS/PDS in the absence of bicarbonate at neutral pH yields Fe^IV^=O_aq_ and not SO_4_^•–^. Furthermore, the results highlight that the presence of HCO_3_^–^ dramatically changed the mechanism and kinetics of Fenton-like processes, here, Fe_aq_^II^ + HSO_5_^–^ and Fe_aq_^II^ + S_2_O_8_^2–^ under most environmental conditions, yielding CO_3_^•–^ radical anions. The reactivity of high-valent iron species in aqueous solution, Fe_aq_^IV^, with pollutants differs from SO_4_^•–^ and OH^•^. This suggests that the antibiotics in the Fe_aq_^II^-activated PMS/PDS are oxidized by Fe_aq_^IV^. However, the presence of HCO_3_^–^ in water generates CO_3_^•–^, which is a weaker oxidizing species and a more selective one. In implementing AOPs under natural environmental conditions, species involved and their effectiveness to degrade different pollutants must be considered carefully.

## Supporting Information Available

Typical kinetic curves for each dependence reactions; derived rate law for the proposed mechanisms; GC determination of methane and ethane; changes in concentrations of PMSO and PMSO_2_ with Fe^II^ in the presence of high bicarbonate systems; pseudo-first-order rate constants of the degradation of SMX and SDM by different persulfate systems (PDF)

## References

[ref1] Lin X.; Choi P. M.; Thompson J.; Reeks T.; Verhagen R.; Tscharke B. J.; O’Malley E.; Shimko K. M.; Guo X.; Thomas K. V.; O’Brien J. W. Systematic Evaluation of the In-Sample Stability of Selected Pharmaceuticals, Illicit Drugs, and Their Metabolites in Wastewater. Environ. Sci. Technol. 2021, 55, 7418–7429. 10.1021/acs.est.1c00396. 34014086

[ref2] Richardson S. D.; Kimura S. Y. Water Analysis: Emerging Contaminants and Current Issues. Anal. Chem. 2020, 92, 473–505. 10.1021/acs.analchem.9b05269. 31825597

[ref3] Keller A. A.; Su Y.; Jassby D. Direct Potable Reuse: Are We Ready? A Review of Technological, Economic, and Environmental Considerations. ACS ES&T Eng. 2022, 2, 273–291. 10.1021/acsestengg.1c00258.

[ref4] Lim S.; Shi J. L.; von Gunten U.; McCurry D. L. Ozonation of Organic Compounds in Water and Wastewater: A Critical Review. Water Res. 2022, 213, 118053 10.1016/j.watres.2022.118053. 35196612

[ref5] Von Gunten U. Oxidation Processes in Water Treatment: Are We on Track?. Environ. Sci. Technol. 2018, 52, 5062–5075. 10.1021/acs.est.8b00586. 29672032

[ref6] Lee J.; Von Gunten U.; Kim J. H. Persulfate-Based Advanced Oxidation: Critical Assessment of Opportunities and Roadblocks. Environ. Sci. Technol. 2020, 54, 3064–3081. 10.1021/acs.est.9b07082. 32062964

[ref7] Wang W.; Chen M.; Wang D.; Yan M.; Liu Z. Different Activation Methods in Sulfate Radical-Based Oxidation for Organic Pollutants Degradation: Catalytic Mechanism and Toxicity Assessment of Degradation Intermediates. Sci. Total Environ. 2021, 772, 145522 10.1016/j.scitotenv.2021.145522. 33571779

[ref8] Moradi S. E.; Dadfarnia S.; Haji Shabani A. M.; Emami S. Removal of Congo Red from Aqueous Solution by Its Sorption onto the Metal Organic Framework MIL-100(Fe): Equilibrium, Kinetic and Thermodynamic Studies. Desalin. Water Treat. 2015, 56, 709–721. 10.1080/19443994.2014.947328.

[ref9] Wen Y.; Huang C. H.; Ashley D. C.; Meyerstein D.; Dionysiou D. D.; Sharma V. K.; Ma X. Visible Light-Induced Catalyst-Free Activation of Peroxydisulfate: Pollutant-Dependent Production of Reactive Species. Environ. Sci. Technol. 2022, 56, 2626–2636. 10.1021/acs.est.1c06696. 35119268

[ref10] Guan Y. H.; Ma J.; Li X. C.; Fang J. Y.; Chen L. W. Influence of PH on the Formation of Sulfate and Hydroxyl Radicals in the UV/Peroxymonosulfate System. Environ. Sci. Technol. 2011, 45, 9308–9314. 10.1021/es2017363. 21999357

[ref11] Olmez-Hanci T.; Arslan-Alaton I. Comparison of Sulfate and Hydroxyl Radical Based Advanced Oxidation of Phenol. Chem. Eng. J. 2013, 224, 10–16. 10.1016/j.cej.2012.11.007.

[ref12] Ike I. A.; Linden K. G.; Orbell J. D.; Duke M. Critical Review of the Science and Sustainability of Persulphate Advanced Oxidation Processes. Chem. Eng. J. 2018, 338, 651–669. 10.1016/j.cej.2018.01.034.

[ref13] Wojnárovits L.; Takács E. Rate Constants of Sulfate Radical Anion Reactions with Organic Molecules: A Review. Chemosphere 2019, 220, 1014–1032. 10.1016/j.chemosphere.2018.12.156. 33395788

[ref14] Zhou Z.; Liu X.; Sun K.; Lin C.; Ma J.; He M.; Ouyang W. Persulfate-Based Advanced Oxidation Processes (AOPs) for Organic-Contaminated Soil Remediation: A Review. Chem. Eng. J. 2019, 372, 836–851. 10.1016/j.cej.2019.04.213.

[ref15] Li J.; Yang L.; Lai B.; Liu C.; He Y.; Yao G.; Li N. Recent Progress on Heterogeneous Fe-Based Materials Induced Persulfate Activation for Organics Removal. Chem. Eng. J. 2021, 414, 128674 10.1016/j.cej.2021.128674.

[ref16] Huang W.; Xiao S.; Zhong H.; Yan M.; Yang X. Activation of Persulfates by Carbonaceous Materials: A Review. Chem. Eng. J. 2021, 418, 129297 10.1016/j.cej.2021.129297.

[ref17] Zheng X.; Niu X.; Zhang D.; Lv M.; Ye X.; Ma J.; Lin Z.; Fu M. Metal-Based Catalysts for Persulfate and Peroxymonosulfate Activation in Heterogeneous Ways: A Review. Chem. Eng. J. 2022, 429, 132323 10.1016/j.cej.2021.132323.

[ref18] Tian D.; Zhou H.; Zhang H.; Zhou P.; You J.; Yao G.; Pan Z.; Liu Y.; Lai B. Heterogeneous Photocatalyst-Driven Persulfate Activation Process under Visible Light Irradiation: From Basic Catalyst Design Principles to Novel Enhancement Strategies. Chem. Eng. J. 2022, 428, 131166 10.1016/j.cej.2021.131166.

[ref19] Gao Y.; Wang Q.; Ji G.; Li A. Degradation of Antibiotic Pollutants by Persulfate Activated with Various Carbon Materials. Chem. Eng. J. 2022, 429, 132387 10.1016/j.cej.2021.132387.

[ref20] Yang J.; Zhu M.; Dionysiou D. D. What Is the Role of Light in Persulfate-Based Advanced Oxidation for Water Treatment?. Water Res. 2021, 189, 116627 10.1016/j.watres.2020.116627. 33221585

[ref21] Anipsitakis G. P.; Dionysiou D. D. Radical Generation by the Interaction of Transition Metals with Common Oxidants. Environ. Sci. Technol. 2004, 38, 3705–3712. 10.1021/es035121o. 15296324

[ref22] Wang Z.; Jiang J.; Pang S.; Zhou Y.; Guan C.; Gao Y.; Li J.; Yang Y.; Qiu W.; Jiang C. Is Sulfate Radical Really Generated from Peroxydisulfate Activated by Iron(II) for Environmental Decontamination?. Environ. Sci. Technol. 2018, 52, 11276–11284. 10.1021/acs.est.8b02266. 30207707

[ref23] Wang Z.; Qiu W.; Pang S.; Zhou Y.; Gao Y.; Guan C.; Jiang J. Further Understanding the Involvement of Fe(IV) in Peroxydisulfate and Peroxymonosulfate Activation by Fe(II) for Oxidative Water Treatment. Chem. Eng. J. 2019, 371, 842–847. 10.1016/j.cej.2019.04.101.

[ref24] Li H.; Shan C.; Li W.; Pan B. Peroxymonosulfate Activation by Iron(III)-Tetraamidomacrocyclic Ligand for Degradation of Organic Pollutants via High-Valent Iron-Oxo Complex. Water Res. 2018, 147, 233–241. 10.1016/j.watres.2018.10.015. 30312796

[ref25] Li H.; Shan C.; Pan B. Fe(III)-Doped g-C3N4 Mediated Peroxymonosulfate Activation for Selective Degradation of Phenolic Compounds via High-Valent Iron-Oxo Species. Environ. Sci. Technol. 2018, 52, 2197–2205. 10.1021/acs.est.7b05563. 29373017

[ref26] Goldstein S.; Meyerstein D.; Czapski G. The Fenton Reagents. Free Radical Biol. Med. 1993, 15, 435–445. 10.1016/0891-5849(93)90043-T. 8225025

[ref27] Meyerstein D. What Are the Oxidizing Intermediates in the Fenton and Fenton-like Reactions? A Perspective. Antioxidants 2022, 11, 1368 10.3390/antiox11071368. 35883862 PMC9312186

[ref28] Masarwa M.; Cohen H.; Meyerstein D.; Hickman D. L.; Bakac A.; Espenson J. H. Reactions of Low-Valent Transition-Metal Complexes with Hydrogen Peroxide. Are They “Fenton-like” or Not? 1. The Case of Cu+aq and Cr2+aq. J. Am. Chem. Soc. 1988, 110, 4293–4297. 10.1021/ja00221a031.

[ref29] Liu J.; Peng C.; Shi X. Preparation, Characterization, and Applications of Fe-Based Catalysts in Advanced Oxidation Processes for Organics Removal: A Review. Environ. Pollut. 2022, 293, 118565 10.1016/j.envpol.2021.118565. 34822943

[ref30] Hou K.; Pi Z.; Yao F.; Wu B.; He L.; Li X.; Wang D.; Dong H.; Yang Q. A Critical Review on the Mechanisms of Persulfate Activation by Iron-Based Materials: Clarifying Some Ambiguity and Controversies. Chem. Eng. J. 2021, 407, 127078 10.1016/j.cej.2020.127078.

[ref31] Duan J.; Pang S.; Wang Z.; Zhou Y.; Gao Y.; Li J.; Guo Q.; Jiang J. Hydroxylamine Driven Advanced Oxidation Processes for Water Treatment: A Review. Chemosphere 2021, 262, 128390 10.1016/j.chemosphere.2020.128390. 33182154

[ref32] Karim A. V.; Jiao Y.; Zhou M.; Nidheesh P. V. Iron-Based Persulfate Activation Process for Environmental Decontamination in Water and Soil. Chemosphere 2021, 265, 129057 10.1016/j.chemosphere.2020.129057. 33272667

[ref33] Kiejza D.; Kotowska U.; Polińska W.; Karpińska J. Peracids - New Oxidants in Advanced Oxidation Processes: The Use of Peracetic Acid, Peroxymonosulfate, and Persulfate Salts in the Removal of Organic Micropollutants of Emerging Concern – A Review. Sci. Total Environ. 2021, 790, 148195 10.1016/j.scitotenv.2021.148195. 34380254

[ref34] Zhou H.; Zhang H.; He Y.; Huang B.; Zhou C.; Yao G.; Lai B. Critical Review of Reductant-Enhanced Peroxide Activation Processes: Trade-off between Accelerated Fe3+/Fe2+ Cycle and Quenching Reactions. Appl. Catal., B 2021, 286, 119900 10.1016/j.apcatb.2021.119900.

[ref35] Dong H.; Xu Q.; Lian L.; Li Y.; Wang S.; Li C.; Guan X. Degradation of Organic Contaminants in the Fe(II)/Peroxymonosulfate Process under Acidic Conditions: The Overlooked Rapid Oxidation Stage. Environ. Sci. Technol. 2021, 55, 15390–15399. 10.1021/acs.est.1c04563. 34730346

[ref36] Patra S. G.; Mizrahi A.; Meyerstein D. The Role of Carbonate in Catalytic Oxidations. Acc. Chem. Res. 2020, 53, 2189–2200. 10.1021/acs.accounts.0c00344. 32975405 PMC7584338

[ref37] Wang J.; Wang S. Effect of Inorganic Anions on the Performance of Advanced Oxidation Processes for Degradation of Organic Contaminants. Chem. Eng. J. 2021, 411, 128392 10.1016/j.cej.2020.128392.

[ref38] Meyerstein D. Re-Examining Fenton and Fenton-like Reactions. Nat. Rev. Chem. 2021, 5, 595–597. 10.1038/s41570-021-00310-4. 37118415

[ref39] Illés E.; Mizrahi A.; Marks V.; Meyerstein D. Carbonate-Radical-Anions, and Not Hydroxyl Radicals, Are the Products of the Fenton Reaction in Neutral Solutions Containing Bicarbonate. Free Radical Biol. Med. 2019, 131, 1–6. 10.1016/j.freeradbiomed.2018.11.015. 30458276

[ref40] Illés E.; Patra S. G.; Marks V.; Mizrahi A.; Meyerstein D. The FeII(Citrate) Fenton Reaction under Physiological Conditions. J. Inorg. Biochem. 2020, 206, 111018 10.1016/j.jinorgbio.2020.111018. 32050088

[ref41] Burg A.; Shamir D.; Shusterman I.; Kornweitz H.; Meyerstein D. The Role of Carbonate as a Catalyst of Fenton-like Reactions in AOP Processes: CO3– as the Active Intermediate. Chem. Commun. 2014, 50, 13096–13099. 10.1039/c4cc05852f. 25223650

[ref42] Yang Y.; Lu X.; Jiang J.; Ma J.; Liu G.; Cao Y.; Liu W.; Li J.; Pang S.; Kong X.; Luo C. Degradation of Sulfamethoxazole by UV, UV/H2O2 and UV/Persulfate (PDS): Formation of Oxidation Products and Effect of Bicarbonate. Water Res. 2017, 118, 196–207. 10.1016/j.watres.2017.03.054. 28431352

[ref43] Zilberg S.; Mizrahi A.; Meyerstein D.; Kornweitz H. Carbonate and Carbonate Anion Radicals in Aqueous Solutions Exist as CO3(H2O)62- and CO3(H2O)6- Respectively: The Crucial Role of the Inner Hydration Sphere of Anions in Explaining Their Properties. Phys. Chem. Chem. Phys. 2018, 20, 9429–9435. 10.1039/c7cp08240a. 29565065

[ref44] Armstrong D. A.; Huie R. E.; Koppenol W. H.; Lymar S. V.; Merenyi G.; Neta P.; Ruscic B.; Stanbury D. M.; Steenken S.; Wardman P. Standard Electrode Potentials Involving Radicals in Aqueous Solution: Inorganic Radicals (IUPAC Technical Report). Pure Appl. Chem. 2015, 87, 1139–1150. 10.1515/pac-2014-0502.

[ref45] Bachi A.; Dalle-Donne I.; Scaloni A. Redox Proteomics: Chemical Principles, Methodological Approaches and Biological/Biomedical Promises. Chem. Rev. 2013, 113, 596–698. 10.1021/cr300073p. 23181411

[ref46] Huie R. E. NDRL/NIST Solution Kinetics Database on the Web; NIST: 2003.

[ref47] Mizrahi A.; Zilbermann I.; Maimon E.; Cohen H.; Meyerstein D. Different Oxidation Mechanisms of MnII(Polyphosphate)n by the Radicals And. J. Coord. Chem. 2016, 69, 1709–1721. 10.1080/00958972.2016.1190451.

[ref48] Bataineh H.; Pestovsky O.; Bakac A. PH-Induced Mechanistic Changeover from Hydroxyl Radicals to Iron(Iv) in the Fenton Reaction. Chem. Sci. 2012, 3, 1594 10.1039/c2sc20099f.

[ref49] Pestovsky O.; Stoian S.; Bominaar E. L.; Shan X.; Münck E.; Que L.; Bakac A. Aqueous FeIV =O: Spectroscopic Identification and Oxo-Group Exchange. Angew. Chem. 2005, 117, 7031–7034. 10.1002/ange.200502686. 16206322

[ref50] Luo C.; Feng M.; Sharma V. K.; Huang C. H. Oxidation of Pharmaceuticals by Ferrate(VI) in Hydrolyzed Urine: Effects of Major Inorganic Constituents. Environ. Sci. Technol. 2019, 53, 5272–5281. 10.1021/acs.est.9b00006. 30933490

[ref51] Kornweitz H.; Burg A.; Meyerstein D. Plausible Mechanisms of the Fenton-like Reactions, M = Fe(II) and Co(II), in the Presence of RCO2- Substrates: Are OH• Radicals Formed in the Process?. J. Phys. Chem. A 2015, 119, 4200–4206. 10.1021/jp512826f. 25891820

[ref52] Bühl M.; Dabell P.; Manley D. W.; McCaughan R. P.; Walton J. C. Bicarbonate and Alkyl Carbonate Radicals: Structural Integrity and Reactions with Lipid Components. J. Am. Chem. Soc. 2015, 137, 16153–16162. 10.1021/jacs.5b10693. 26623482

[ref53] Medinas D. B.; Cerchiaro G.; Trindade D. F.; Augusto O. The Carbonate Radical and Related Oxidants Derived from Bicarbonate Buffer. IUBMB Life 2007, 59, 255–262. 10.1080/15216540701230511. 17505962

[ref54] Cope V. W.; Huffman M. Z.; Chen S. N. Reactivity of the Carbonate Radical toward Metal Complexes in Aqueous Solution. J. Phys. Chem. A 1978, 82, 2665–2669. 10.1021/j100514a007.

[ref55] Fouillac C.; Criaud A. Carbonate and Bicarbonate Trace Metal Complexes: Critical Reevaluation of Stability Constants. Geochem. J. 1984, 18, 297–303. 10.2343/geochemj.18.297.

[ref56] Rachmilovich-Calis S.; Masarwa A.; Meyerstein N.; Meyerstein D. The Effect of Pyrophosphate, Tripolyphosphate and ATP on the Rate of the Fenton Reaction. J. Inorg. Biochem. 2011, 105, 669–674. 10.1016/j.jinorgbio.2011.01.009. 21450270

[ref57] Wang Z.; Qiu W.; Pang S.; Jiang J. Effect of Chelators on the Production and Nature of the Reactive Intermediates Formed in Fe(II) Activated Peroxydisulfate and Hydrogen Peroxide Processes. Water Res. 2019, 164, 114957 10.1016/j.watres.2019.114957. 31421513

[ref58] Wang Z.; Qiu W.; Pang S.; Gao Y.; Zhou Y.; Cao Y.; Jiang J. Relative Contribution of Ferryl Ion Species (Fe(IV)) and Sulfate Radical Formed in Nanoscale Zero Valent Iron Activated Peroxydisulfate and Peroxymonosulfate Processes. Water Res. 2020, 172, 115504 10.1016/j.watres.2020.115504. 31981901

[ref59] Wang Z.; Qiu W.; Pang S.; Guo Q.; Guan C.; Jiang J. Aqueous Iron(IV)–Oxo Complex: An Emerging Powerful Reactive Oxidant Formed by Iron(II)-Based Advanced Oxidation Processes for Oxidative Water Treatment. Environ. Sci. Technol. 2022, 56, 1492–1509. 10.1021/acs.est.1c04530. 35007064

[ref60] Herscu-Kluska R.; Masarwa A.; Saphier M.; Cohen H.; Meyerstein D. Mechanism of the Reaction of Radicals with Peroxides and Dimethyl Sulfoxide in Aqueous Solution. Chem. – Eur. J. 2008, 14, 5880–5889. 10.1002/chem.200800218. 18481834

[ref61] Avraham E.; Meyerstein D.; Lerner A.; Yardeni G.; Pevzner S.; Zilbermann I.; Moisy P.; Maimon E.; Popivker I. Reactions of Methyl, Hydroxyl and Peroxyl Radicals with the DOTA Chelating Agent Used in Medical Imaging. Free Radical Biol. Med. 2022, 180, 134–142. 10.1016/j.freeradbiomed.2021.12.313. 34973364

[ref62] Rusonik I.; Polat H.; Cohen H.; Meyerstein D. Reaction of Methyl Radicals with Metal Powders Immersed in Aqueous Solutions. Eur. J. Inorg. Chem. 2003, 2003, 4227–4233. 10.1002/ejic.200300403. 16933942

[ref63] Lerner A.; Kornweitz H.; Zilbermann I.; Yardeni G.; Saphier M.; Bar Ziv R.; Meyerstein D. Radicals in ‘Biologically Relevant’ Concentrations Behave Differently: Uncovering New Radical Reactions Following the Reaction of Hydroxyl Radicals with DMSO. Free Radical Biol. Med. 2021, 162, 555–560. 10.1016/j.freeradbiomed.2020.11.012. 33217506

[ref64] Zhang X.; Feng M.; Luo C.; Nesnas N.; Huang C. H.; Sharma V. K. Effect of Metal Ions on Oxidation of Micropollutants by Ferrate(VI): Enhancing Role of FeIVSpecies. Environ. Sci. Technol. 2021, 55, 623–633. 10.1021/acs.est.0c04674. 33326216

[ref65] Villamena F. A.; Locigno E. J.; Rockenbauer A.; Hadad C. M.; Zweier J. L. Theoretical and Experimental Studies of the Spin Trapping of Inorganic Radicals by 5,5-Dimethyl-1-Pyrroline N-Oxide (DMPO). 2. Carbonate Radical Anion. J. Phys. Chem. A 2007, 111, 384–391. 10.1021/jp065692d. 17214476

[ref66] Davies M. J.; Gilbert B. C.; Stell J. K.; Whitwood A. C. Nucleophilic Substitution Reactions of Spin Adducts. Implications for the Correct Identification of Reaction Intermediates by EPR/Spin Trapping. J. Chem. Soc., Perkin Trans. 2 1992, 3, 333–335. 10.1039/p29920000333.

[ref67] Timmins G. S.; Liu K. J.; Bechara E. J. H.; Kotake Y.; Swartz H. M. Trapping of Free Radicals with Direct in Vivo EPR Detection: A Comparison of 5,5-Dimethyl-1-Pyrroline-N-Oxide and 5-Diethoxyphosphoryl-5-Methyl-1-Pyrroline-N-Oxide as Spin Traps for HO and SO4•–. Free Radical Biol. Med. 1999, 27, 329–333. 10.1016/S0891-5849(99)00049-0. 10468206

[ref68] Kovalakova P.; Cizmas L.; McDonald T. J.; Marsalek B.; Feng M.; Sharma V. K. Occurrence and Toxicity of Antibiotics in the Aquatic Environment: A Review. Chemosphere 2020, 251, 126351 10.1016/j.chemosphere.2020.126351. 32443222

[ref69] Feng M.; Baum J. C.; Nesnas N.; Lee Y.; Huang C. H.; Sharma V. K. Oxidation of Sulfonamide Antibiotics of Six-Membered Heterocyclic Moiety by Ferrate(VI): Kinetics and Mechanistic Insight into SO 2 Extrusion. Environ. Sci. Technol. 2019, 53, 2695–2704. 10.1021/acs.est.8b06535. 30715861

[ref70] Kokoszka K.; Wilk J.; Felis E.; Bajkacz S. Application of UHPLC-MS/MS Method to Study Occurrence and Fate of Sulfonamide Antibiotics and Their Transformation Products in Surface Water in Highly Urbanized Areas. Chemosphere 2021, 283, 131189 10.1016/j.chemosphere.2021.131189. 34153907

