# Supplementary material for: Reaction of FeaqII with Peroxymonosulfate and Peroxydisulfate in the Presence of Bicarbonate: Formation of FeaqIV and Carbonate Radical Anions
Source: Environ Sci Technol. 2023 Apr 13;57(16):6743–53. doi: 10.1021/acs.est.3c00182 (PMC10134498; doi:10.1021/acs.est.3c00182)
Supplement: Supplementary file 1 — es3c00182_si_001.pdf [file es3c00182_si_001.pdf]

# Supporting Information

# Reaction of $Fe^{II}_{aq}$ with Peroxymonosulfate and Peroxydisulfate in Presence of Bicarbonate: Formation of $Fe^{IV}_{aq}$ and Carbonate Radical Anions

Aswin Kottapurath Vijay, [a,b] Vered Marks, [c] Amir Mizrahi, [d] Yinghao Wen,[e] Xingmao Ma,[e] Virender K. Sharma,[f] Dan Meyerstein* [a,b]

[a] Department of Chemical Sciences and The Radical Research Center Ariel University, Ariel 40700, Israel.
E-mail: danm@ariel.ac.il
[b] Chemistry Department, Ben-Gurion University, Beer-Sheva 8410501, Israel
[c] Department of Chemical Sciences, Ariel University, Ariel 40700, Israel
[d] Chemistry Department, Nuclear Research Centre Negev, Beer-Sheva 84190, Israel
[e] Department of Civil and Environmental Engineering, Texas A&M University, College Station, Texas, 77843, United States
[f] Program for the Environment and Sustainability, Department of Environmental and Occupational Health, Texas A&M University, College Station, Texas 77843, United States

**Pages:21, Table:1, Figures:17**

# Table of contents

Table S1 .... 3
Figure S1 .... 4
Figure S2 .... 5
Figure S3 .... 6
Figure S4 .... 7
Figure S5 .... 8
Figure S6 .... 9
Figure S7 .... 10
Figure S8 .... 10
Figure S9 .... 11
Figure S10 .... 12
Figure S11 .... 13
Figure S12 .... 14
Figure S13 .... 15
Text S1 .... 16
Text S2 .... 17
Text S3 .... 18
Figure S14 .... 19
Figure S15 .... 19
Figure S16 .... 20
Figure S17 .... 21

**Table S1.** Six different setups used to determine the degradation efficiencies of SMX and SDM

| # | Treatment | $[PMS]_0$ (mM) | $[PDS]_0$ (mM) | $[Fe^{II}_{aq}]_0$ (mM) | $[bicarbonate]_0$ (mM) |
|---|---|---|---|---|---|
| 1 | PMS alone | 0.04 | 0 | 0 | 0 |
| 2 | $Fe^{II}_{aq}$ - PMS | 0.04 | 0 | 0.2 | 0 |
| 3 | $Fe^{II}_{aq}$ – PMS - bicarbonate | 0.04 | 0 | 0.2 | 0.5 |
| 4 | PDS alone | 0 | 1.0 | 0 | 0 |
| 5 | $Fe^{II}_{aq}$ - PDS | 0 | 1.0 | 2.0 | 0 |
| 6 | $Fe^{II}_{aq}$ – PDS - bicarbonate | 0 | 1.0 | 2.0 | 5.0 |

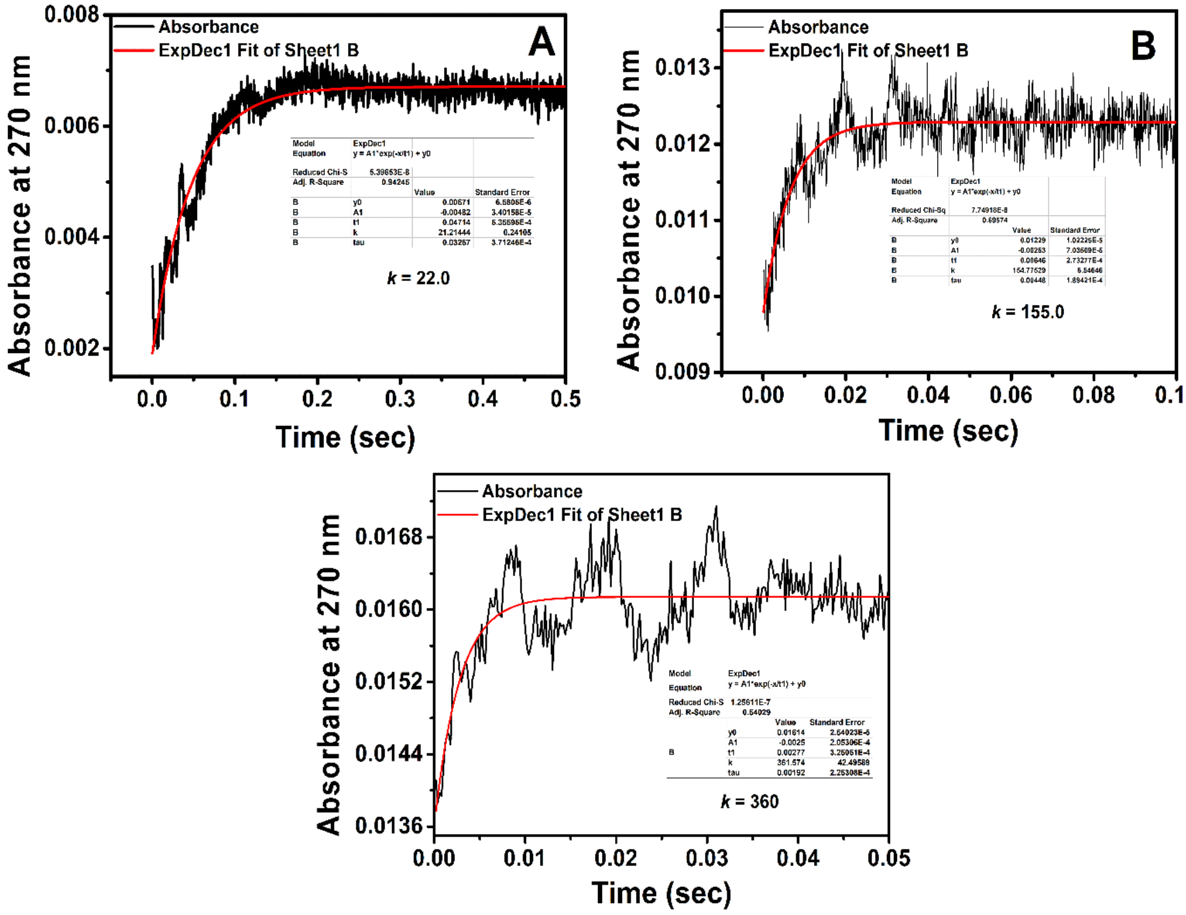


**Figure S1**. Formation of Fe(III) as a function of time during the reaction of $Fe(H_2O)_6^{2+}$ with PMS at different concentrations of PMS while keeping a constant low concentration of $HCO_3^-$. (**A**) [PMS] = 0.05 mM, (**B**) [PMS] = 0.10 mM, and (**C**) [PMS] = 0.20 mM. (Experimental initial concentrations in the Fenton solutions: )$[Fe^{II}]$ = 0.020 mM, $[HCO_3^-]$ = 0.50 mM.

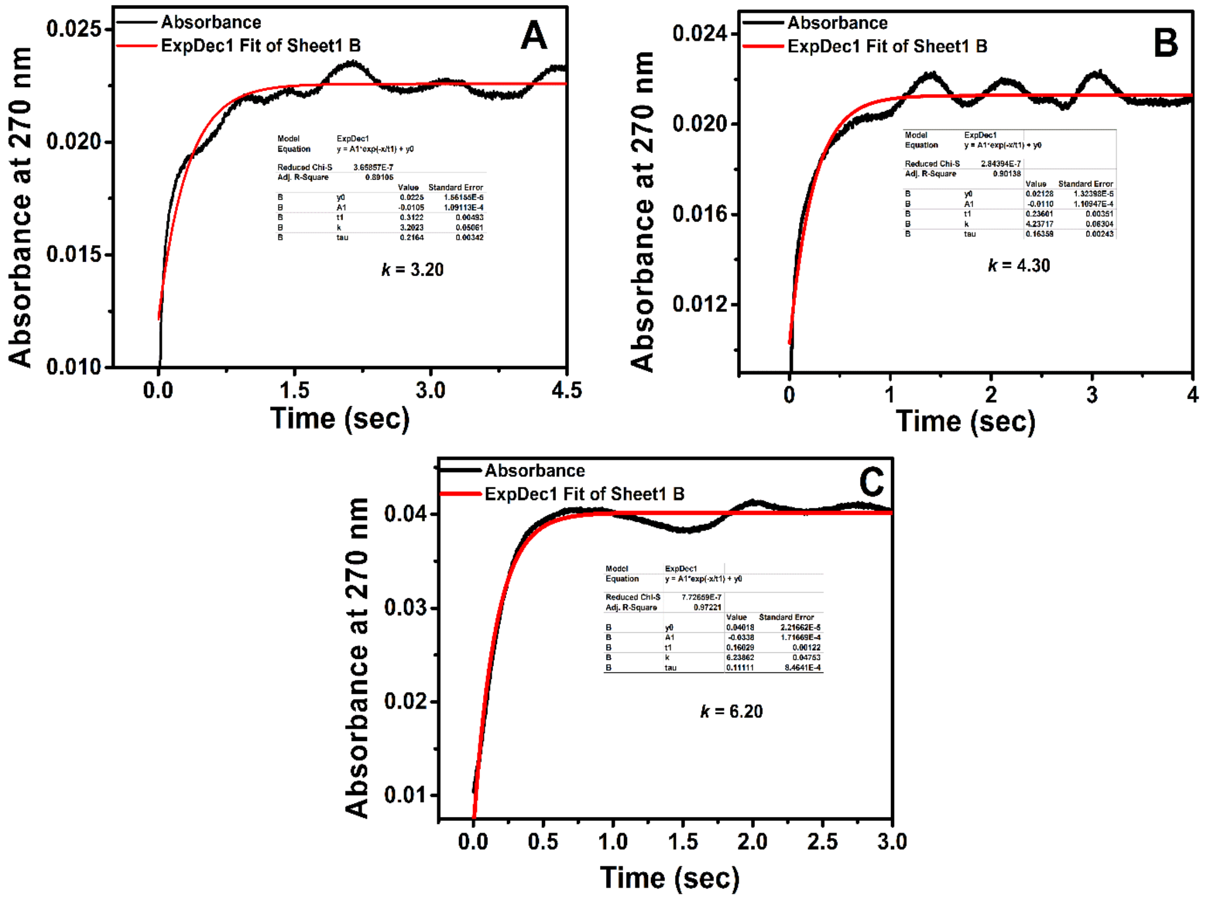


**Figure S2**. Formation of Fe(III) as a function of time during the reaction of $Fe(H_2O)_6^{2+}$ with PDS at different concentrations of PDS while keeping a constant concentration of $HCO_3^-$. (**A**) [PDS] = 1.0 mM, (**B**) [PDS] = 2.0 mM, and (**C**) [PDS] = 3.0 mM. (Experimental initial concentrations in the Fenton solutions: $[Fe^{II}]$ = 0.10 mM, $[HCO_3^-]$ = 8.0 mM).

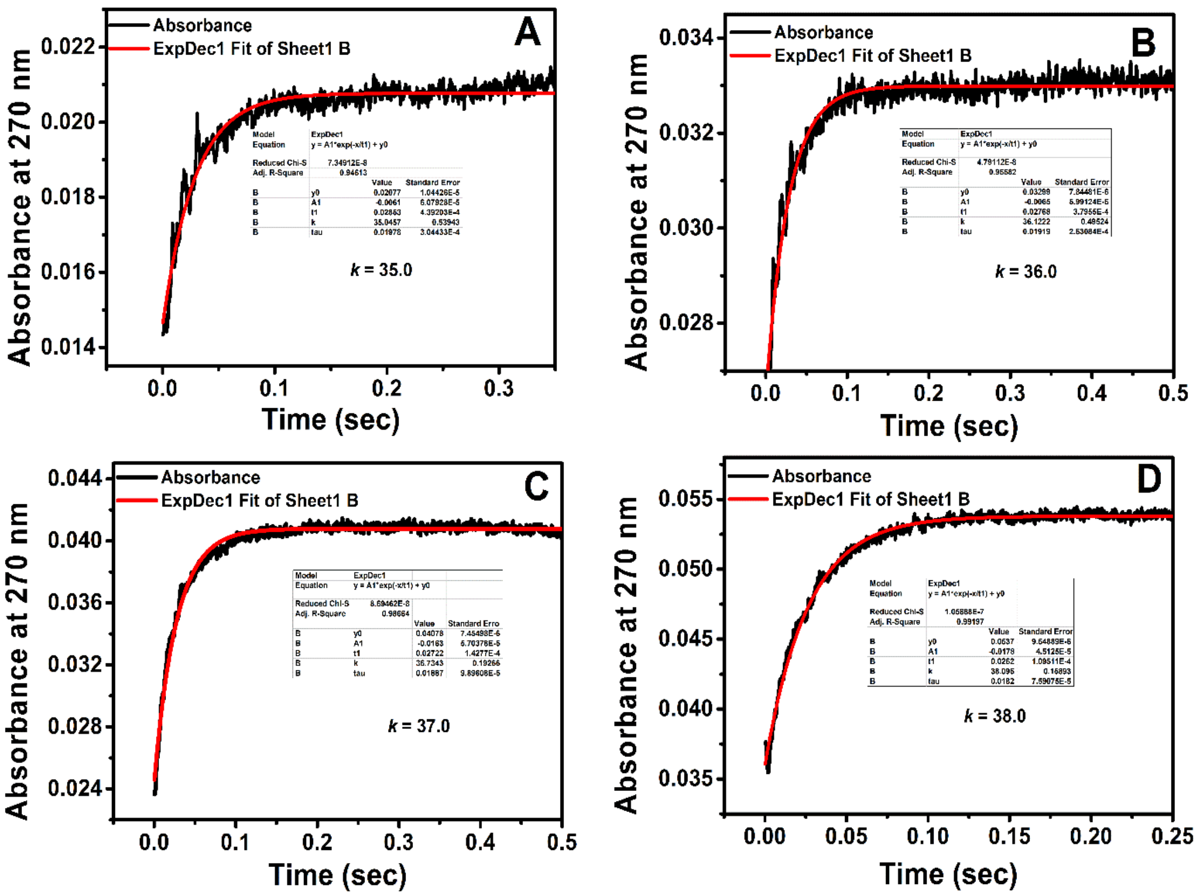


**Figure S3**. Typical kinetic curves of the formation of Fe(III) during the reaction of $Fe(H_2O)_6^{2+}$ with PMS at different concentrations of $Fe^{II}_{aq}$ under a constant concentration of low $HCO_3^-$. (**A**) [$Fe^{II}_{aq}$= 0.02 mM, (**B**) [$Fe^{II}_{aq}$] = 0.04 mM, (**C**) [$Fe^{II}_{aq}$] = 0.06 mM, and (**D**) [$Fe^{II}_{aq}$] = 0.08 mM (Experimental initial concentrations in the Fenton solutions: [PMS] = 0.10 mM and [$HCO_3^-$]= 0.30 mM).

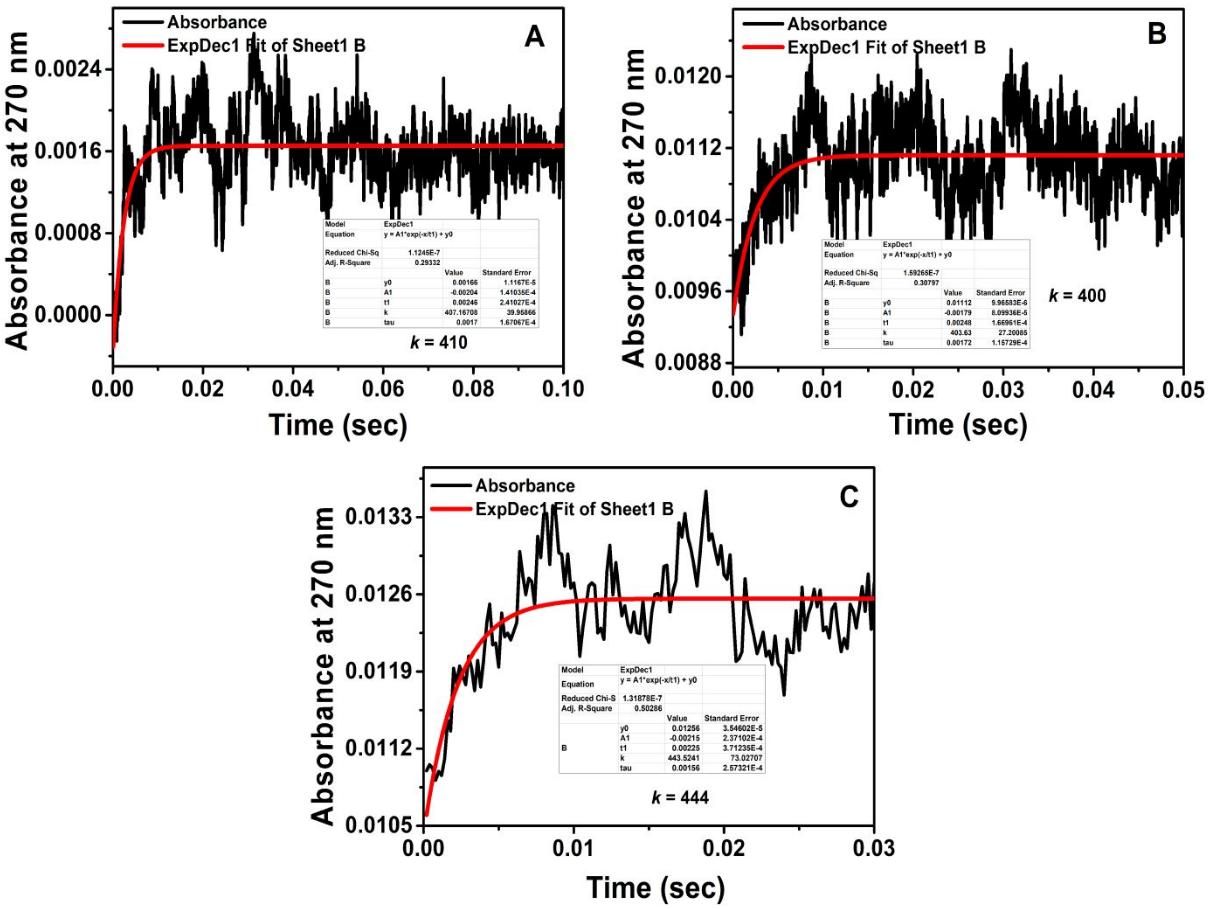


**Figure S4**. Typical kinetic curves of the formation of Fe(III) during the reaction of $Fe(H_2O)_6^{2+}$ with PMS at different concentrations of $Fe^{II}_{aq}$ under a constant concentration of high $HCO_3^-$. (**A**) [$Fe^{II}_{aq}$= 0.02 mM, (**B**) [$Fe^{II}_{aq}$] = 0.04 mM and (**C**) [$Fe^{II}_{aq}$] = 0.08 mM (Experimental initial concentrations in the Fenton solutions: [PMS] = 0.10 mM and [$HCO_3^-$]= 0.60 mM).

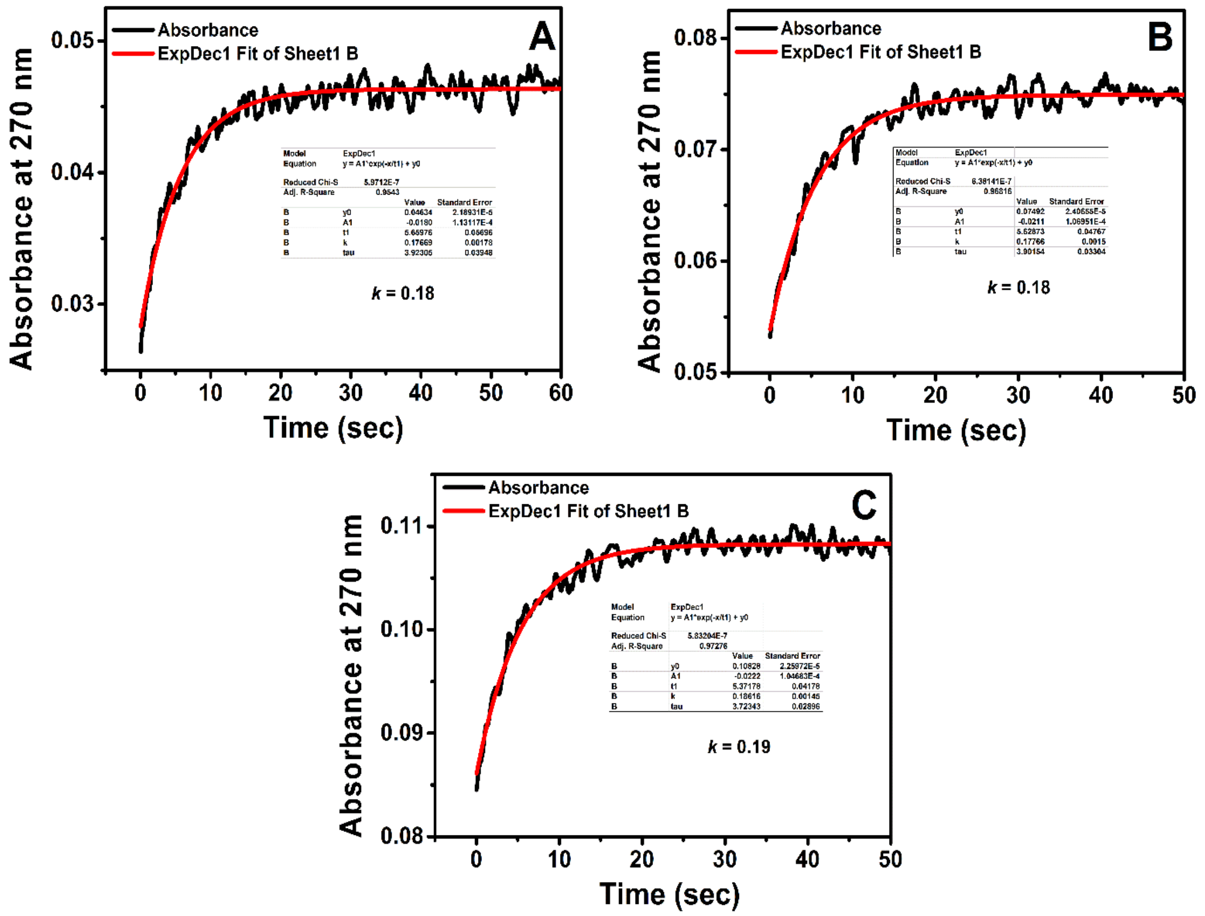


**Figure S5**. Typical kinetic curves of the formation of Fe(III) during the reaction of $Fe(H_2O)_6^{2+}$ with PDS at different concentrations of $Fe^{II}_{aq}$ under a constant concentration of low $HCO_3^-$. (**A**) [$Fe^{II}_{aq}$] = 0.10 mM, (**B**) [$Fe^{II}_{aq}$] = 0.20 mM, and (**C**) [$Fe^{II}_{aq}$] = 0.40 mM $Fe^{II}_{aq}$. (Experimental initial concentrations in the Fenton solutions: [PDS] = 1.0 mM, [$HCO_3^-$] = 2.0 mM)

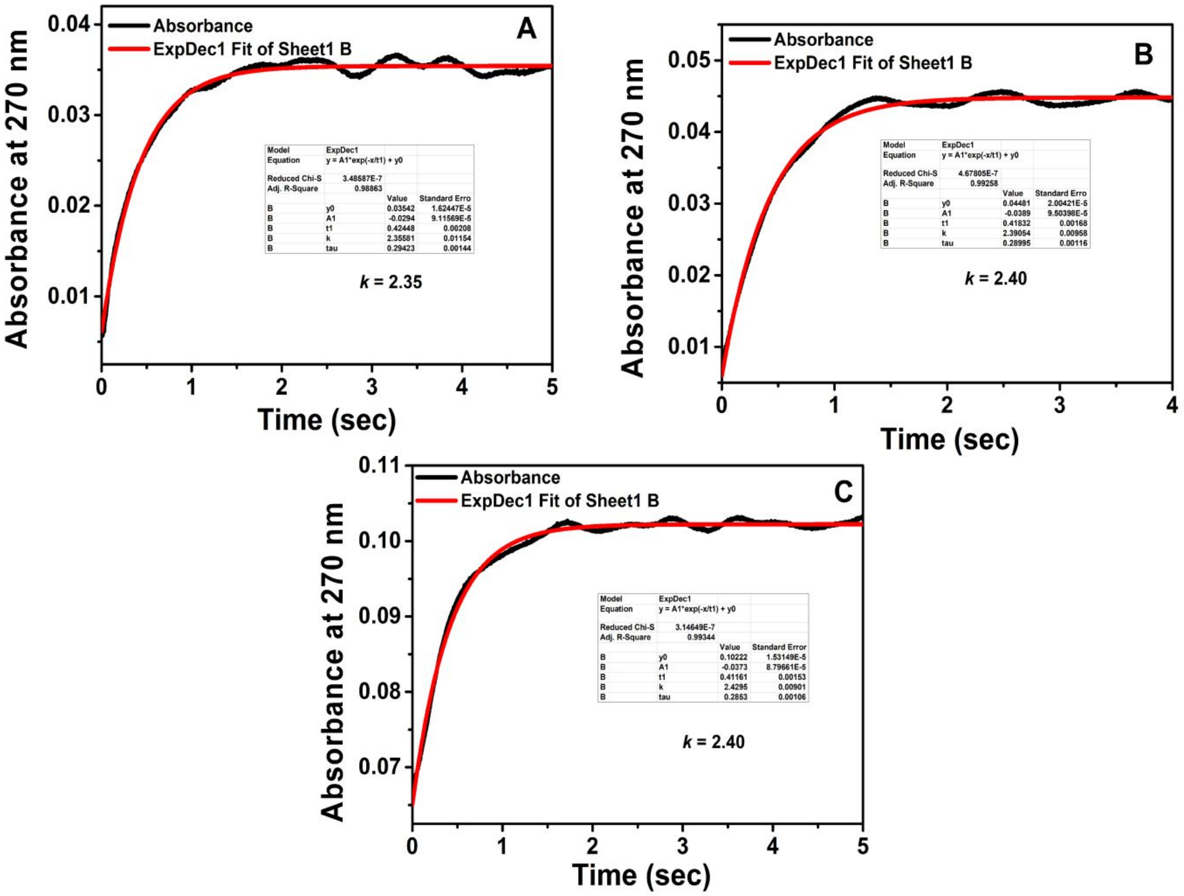


**Figure S6**. Typical kinetic curves of the formation of Fe(III) during the reaction of $Fe(H_2O)_6^{2+}$ with PDS at different concentrations of $Fe^{II}_{aq}$ under a constant concentration of high $HCO_3^-$. (**A**) [$Fe^{II}_{aq}$] = 0.10 mM, (**B**) [$Fe^{II}_{aq}$] = 0.20 mM, and (**C**) [$Fe^{II}_{aq}$] = 0.40 mM $Fe^{II}_{aq}$. (Experimental initial concentrations in the Fenton solutions: [PDS] = 1.0 mM, [$HCO_3^-$] = 5.0 mM)

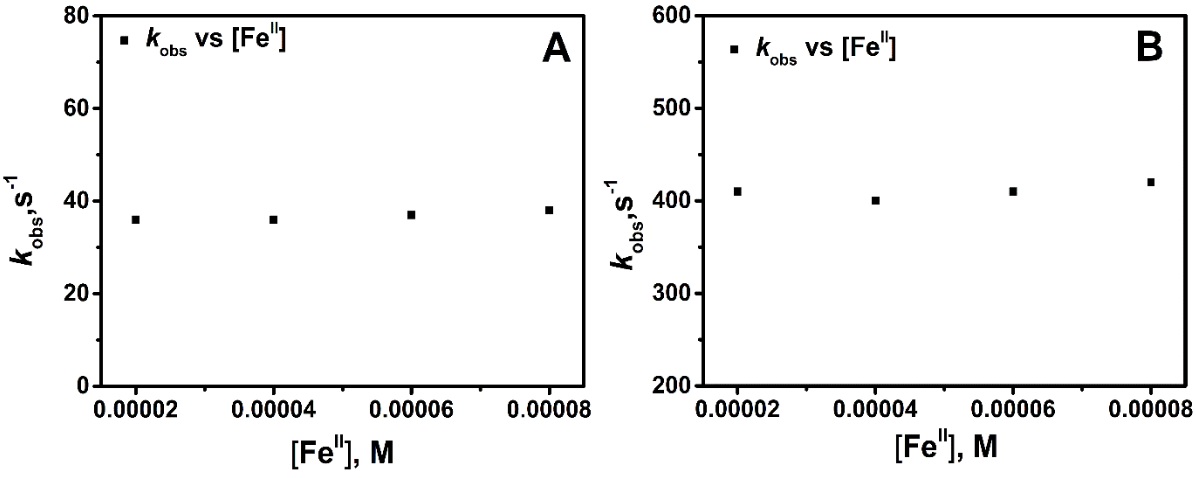


**Figure S7**. Dependence of $k_{obs}$ at pH 7.40 on the concentrations of $Fe^{II}_{aq}$, at a constant concentration of PMS **(A)** [$HCO_3^-$ ] = 0.30 mM (Low [$HCO_3^-$]) and **(B)** [$HCO_3^-$] = 0.60 mM (High [$HCO_3^-$]). Here in both cases, [PMS] = 0.10 mM

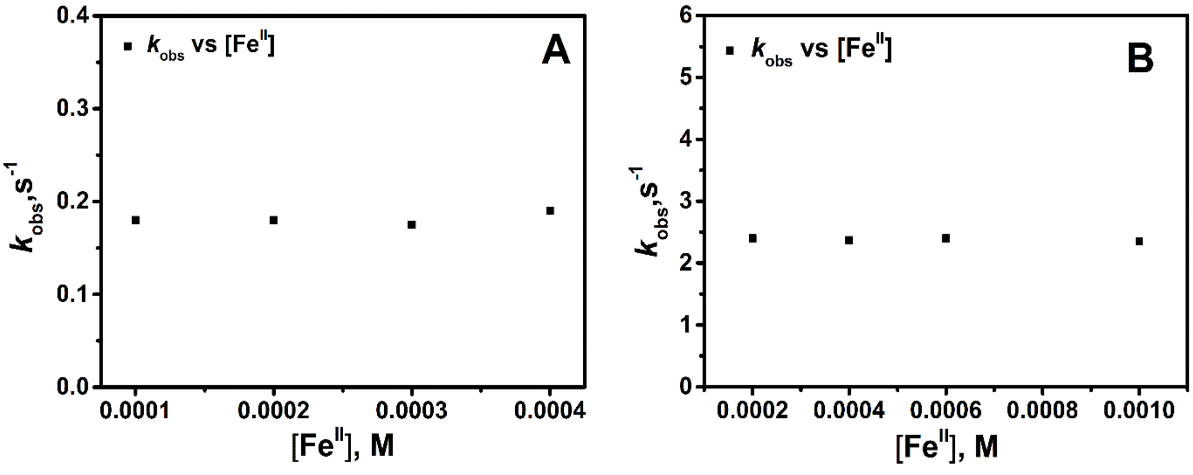


**Figure S8**. Dependence of $k_{obs}$ at pH 7.40 on the concentrations of $Fe^{II}_{aq}$, at a constant concentration of PDS **(A)** [$HCO_3^-$ ] = 2.0 mM (Low [$HCO_3^-$]) and **(B)** [$HCO_3^-$] = 5.0 mM (High [$HCO_3^-$]). Here in both cases, [PDS] = 1.0 mM

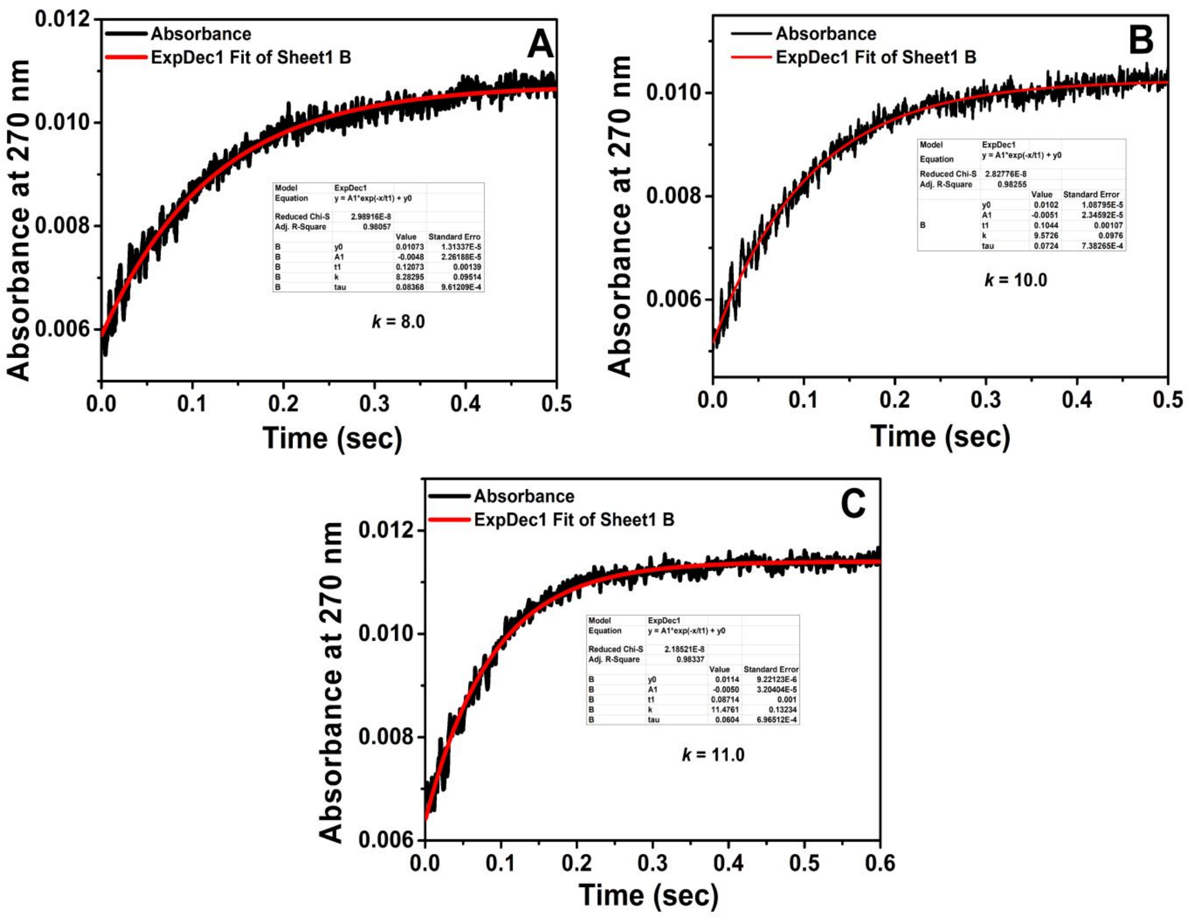


**Figure S9**. Kinetic curves of the formation of Fe(III) during the reaction of $Fe(H_2O)_6^{2+}$ with PMS in the absence of bicarbonate at different pH's (A) pH = 5.80, (B) pH = 7.0 and (C) pH = 8.40. (Experimental initial concentrations in the Fenton solutions: [PMS] = 0.20 mM, $[Fe^{II}_{aq}]$ = 0.020 mM)

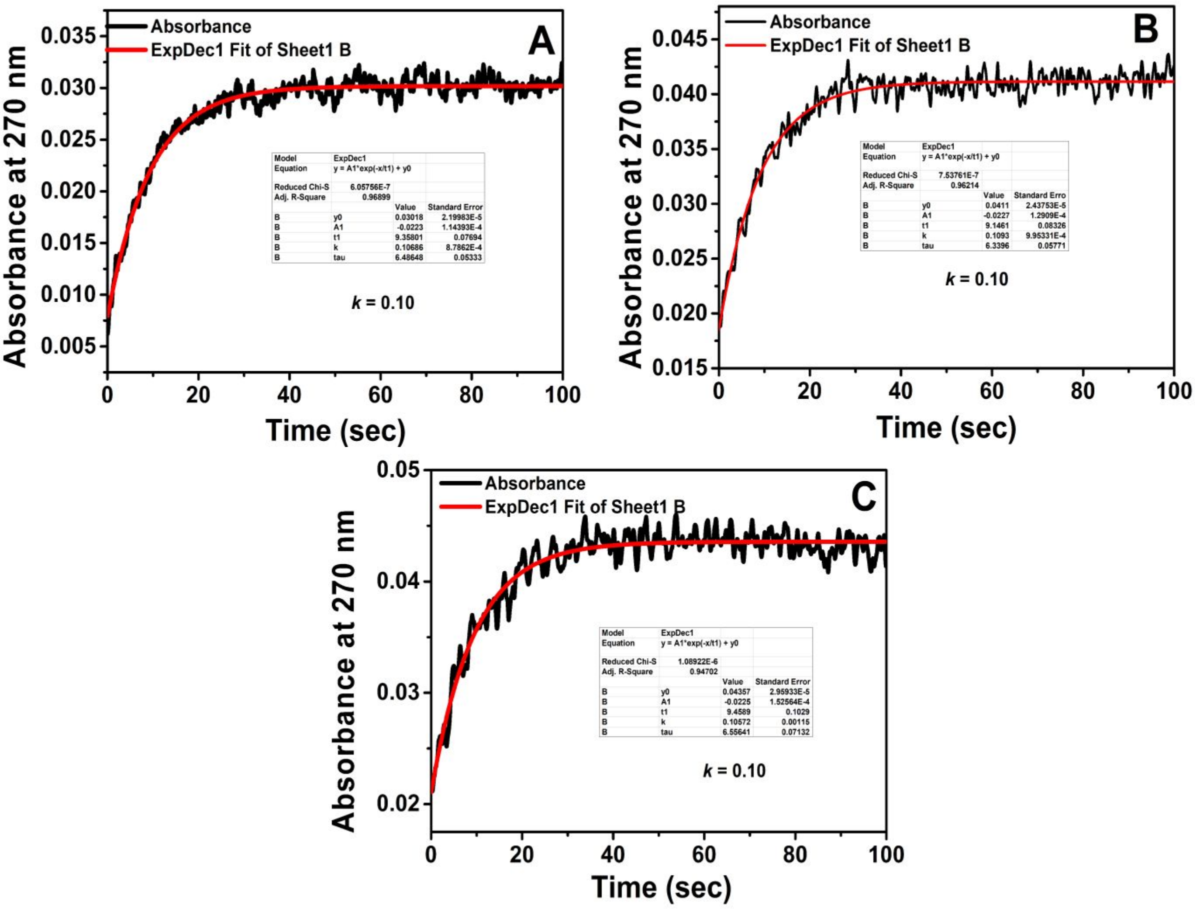


**Figure S10**. Kinetic curves of the formation of Fe(III) during the reaction of $Fe(H_2O)_6^{2+}$ with PDS in the absence of bicarbonate at different pH's (A) pH = 5.80, (B) pH = 7.0 and (C) pH = 8.40. (Experimental initial concentrations in the Fenton solutions: [PDS] = 1.0 mM, $[Fe^{II}_{aq}]$ = 0.10 mM)

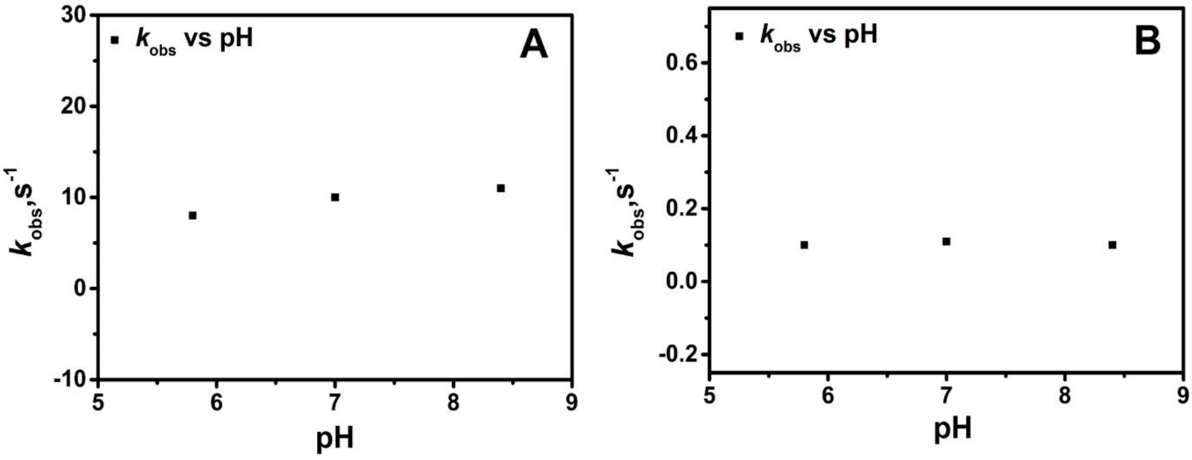


**Figure S11**. Dependence of $k_{obs}$ on the pH in the absence of bicarbonate at a constant concentration of (**A**) PMS ([PMS] = 0.20 mM, [$Fe^{II}_{aq}$] = 0.020 mM) and (**B**) PDS ([PDS] = 1.0 mM, [$Fe^{II}_{aq}$] = 0.10 mM)

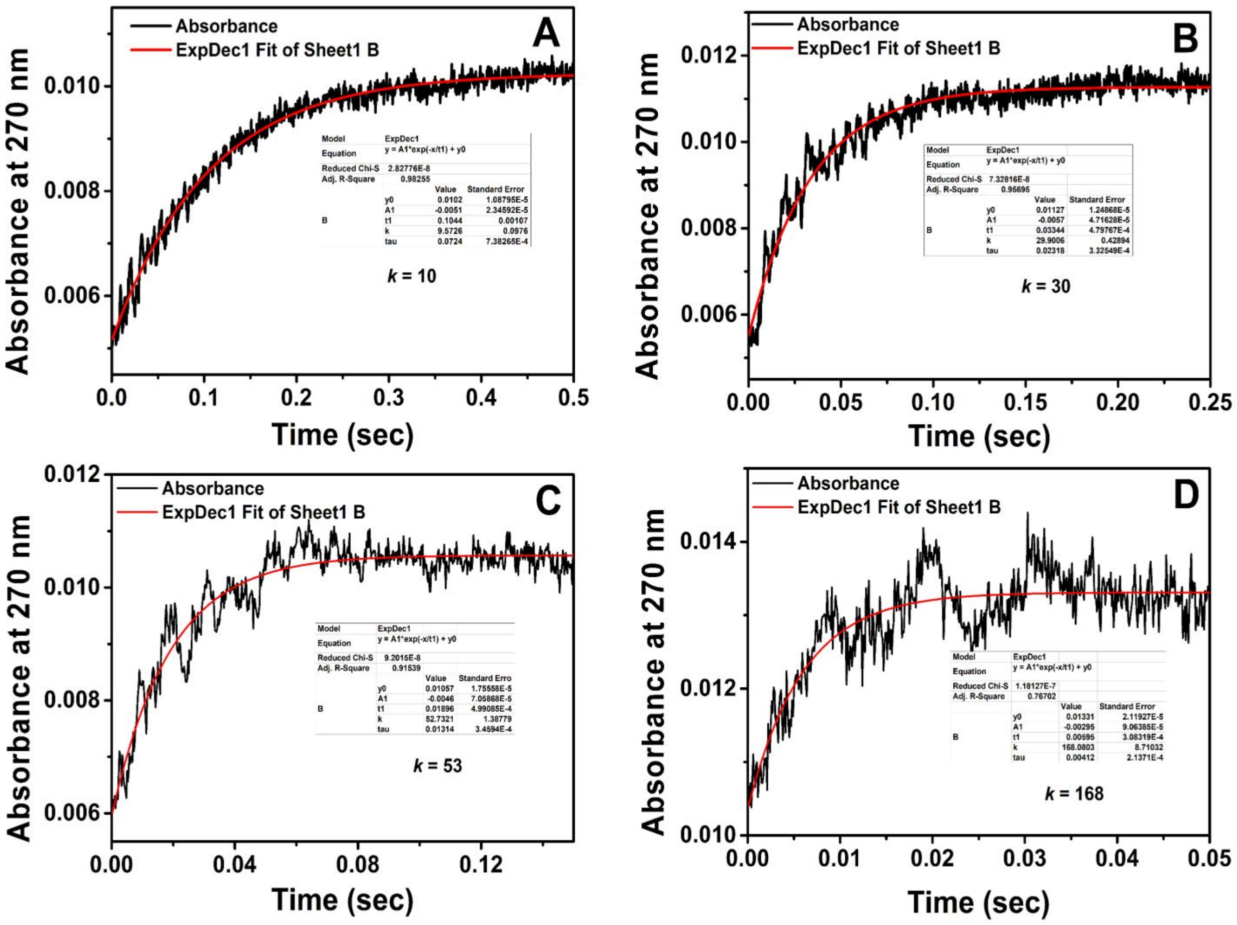


**Figure S12**. Typical kinetic curve of the reaction of $Fe(H_2O)_6^{2+}$ with PMS in presence of excess PMS taken by stopped flow instrument with different concentration of (A) 0 mM $HCO_3^-$ (B) 0.10 mM $HCO_3^-$ (C) 0.20 mM $HCO_3^-$ and (D) 0.40 mM $HCO_3^-$ .The initial concentrations in the Fenton solutions are [$Fe^{II}$] = 0.020 mM, [PMS] = 0.20 mM. The data of the conventional spectrophotometric measurements were analyzed graphically, and the *k* values were determined by fitting single exponential curves to the absorbance *vs*. time plots.

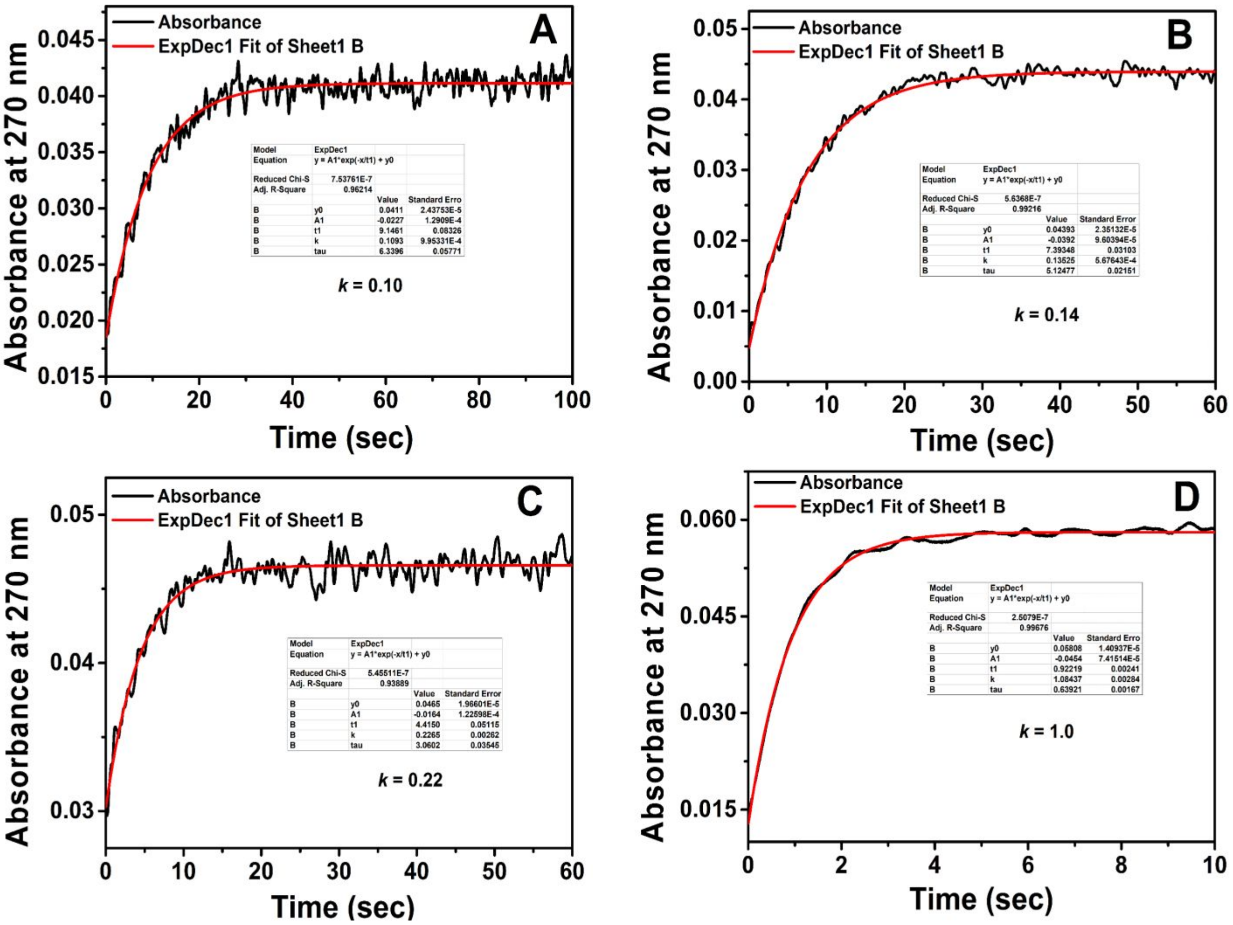


**Figure S13.** Typical kinetic curve of the reaction of $Fe(H_2O)_6^{2+}$ with PDS in presence of excess PDS taken by stopped flow instrument with different concentration of (**A**) 0 mM $HCO_3^-$ (**B**) 1.0 mM $HCO_3^-$ (**C**) 2.0 mM $HCO_3^-$ and (**D**) 3.0 mM $HCO_3^-$ .The initial concentrations in the Fenton solutions are $[Fe^{II}]$ = 0.10 mM, [PDS] = 1.0 mM.

**Text S1:**

***Mechanism I:***

***Small slope***

$Fe(H_2O)_6^{2+} + HSO_5^- \rightleftharpoons (H_2O)_5Fe^{II}(O_2SO_3)\ /(H_2O)_3Fe^{II}(O_2SO_3) + H_3O^+$ (8)

$(H_2O)_5Fe^{II}(O_2SO_3)\ /(H_2O)_3Fe^{II}(O_2SO_3) + HCO_3^- \rightarrow Fe^{III}_{aq} + CO_3^{\cdot -} + SO_4^{2-}$

(small slope) (9)

Since the rate determining step (rds) is Eq. (9), the overall rate law for this reaction is as follows:

rate = $k_9\ [(H_2O)_5Fe^{II}(O_2SO_3)\ /(H_2O)_3Fe^{II}(O_2SO_3)]\ [HCO_3^-]$ (9'')

Since $(H_2O)_5Fe^{II}(O_2SO_3)\ /(H_2O)_3Fe^{II}(O_2SO_3)$ is an intermediate, from eqtn (8),

$k_8\ [Fe(H_2O)_6^{2+}]\ [HSO_5^-] = k_{-8}\ [(H_2O)_5Fe^{II}(O_2SO_3)\ /(H_2O)_3Fe^{II}(O_2SO_3)]$ (8'')

This equation may also be written in the following alternative way,

$[(H_2O)_5Fe^{II}(O_2SO_3)\ /(H_2O)_3Fe^{II}(O_2SO_3)] = K_8\ [Fe(H_2O)_6^{2+}]\ [HSO_5^-]$

Eq. (9'') may be rewritten as,

**rate = $\mathbf{k_9\, K_8\ [Fe(H_2O)_6^{2+}]\ [HSO_5^-]\ [HCO_3^-]}$**

***Large slope***

$Fe(H_2O)_6^{2+} + HCO_3^- \rightleftharpoons (H_2O)_3Fe(CO_3) + H_3O^+ + 2H_2O$ (10)

$(H_2O)_3Fe(CO_3) + HSO_5^-/S_2O_8^{2-} \rightarrow Fe^{III}_{aq} + CO_3^{\cdot -} + SO_4^{2-}/2SO_4^{2-}$

(large slope) (10')

The overall rate law of this reaction is as follows:

$r = k_{10'}\ [(H_2O)_3Fe(CO_3)]\ [HSO_5^-/S_2O_8^{2-}]$ (10'')

Here, $(H_2O)_3Fe(CO_3)$ is the intermediate formed. Rearranging the equilibrium rate equation (10), the $[(H_2O)_3Fe(CO_3)]$ is given by,

$[(H_2O)_3Fe(CO_3)] = K_{10}\ [Fe(H_2O)_6^{2+}]\ [HCO_3^-]$

The overall rate of this reaction can be expressed by rearranging the Eq. (10'') as

rate = $\mathbf{k_{10'}\, K_{10}\ [Fe(H_2O)_6^{2+}]\ [HCO_3^-]\ [HSO_5^-/S_2O_8^{2-}]}$

## Text S2

### *Mechanism II:*

***Small slope***

$Fe(H_2O)_6^{2+} + HCO_3^{-} \rightleftharpoons (H_2O)_3Fe(CO_3) + H_3O^{+} + 2H_2O$ (11)

$(H_2O)_3Fe(CO_3) + HSO_5^{-}/S_2O_8^{2-} \rightarrow Fe^{III}_{aq} + CO_3^{\cdot -} + SO_4^{2-}/2SO_4^{2-}$

(small slope) (12)

The overall rate law for this reaction (rds = (12)) is as follows

$r = k_{12}\, [(H_2O)_3Fe(CO_3)]\, [HSO_5^{-}/S_2O_8^{2-}]$ (12')

Here, $(H_2O)_3Fe(CO_3)$ is an intermediate

From (11),

$r_{forward} = r_{backward}$

$k_{11}\, [Fe(H_2O)_6^{2+}]\, [HCO_3^{-}] = k_{-11}\, [(H_2O)_3Fe(CO_3)]$ (12'')

$[(H_2O)_3Fe(CO_3)] = K_{11}[Fe(H_2O)_6^{2+}]\, [HCO_3^{-}]$

Rearrange the Eq. (12') by substituting the value for $[(H_2O)_3Fe(CO_3)]$,

**rate = $k_{12}\, K_{11}\, [Fe(H_2O)_6^{2+}]\, [HCO_3^{-}]\, [HSO_5^{-}/S_2O_8^{2-}]$**

***Large slope***

$(H_2O)_3Fe(CO_3) + HSO_5^{-}/S_2O_8^{2-} \rightleftharpoons$

$(H_2O)Fe(CO_3)(O_2SO_3)/(H_2O)Fe(CO_3)(S_2O_8) + H_3O^{+}$ (13)

$(H_2O)Fe(CO_3)(O_2SO_3)/(H_2O)Fe(CO_3)(S_2O_8) + HCO_3^{-} \rightleftharpoons$

$Fe^{III}(CO_3)_{aq} + CO_3^{\cdot -} + SO_4^{2-}/2SO_4^{2-}$ (large slope) (14)

Overall rate is as followed:

$r = k_{14}\, [(H_2O)Fe(CO_3)(O_2SO_3)/(H_2O)Fe(CO_3)(S_2O_8)]\, [HCO_3^{-}]$ (14')

Here $(H_2O)Fe(CO_3)(O_2SO_3)/(H_2O)Fe(CO_3)(S_2O_8)$ is an intermediate

Hence, from Eq. (13)

$[(H_2O)Fe(CO_3)(O_2SO_3)/(H_2O)Fe(CO_3)(S_2O_8)] = K_{13}\, [(H_2O)_3Fe(CO_3)]\, [HSO_5^{-}/S_2O_8^{2-}]$

(14'')

Substitute the $[(H_2O)_3Fe(CO_3)]$ and $[(H_2O)Fe(CO_3)(O_2SO_3)/(H_2O)Fe(CO_3)(S_2O_8)]$ in (14')

**rate = $k_{14}K_{13}K_{11}\, [Fe(H_2O)_6^{2+}]\, [HCO_3^{-}]^2\, [HSO_5^{-}/S_2O_8^{2-}]$**

**<u>Text S3</u>**

***Mechanism III:***

***Small slope***

$Fe(H_2O)_6^{2+} + HCO_3^- \rightleftharpoons (H_2O)_3Fe(CO_3) + H_3O^+ + 2H_2O$ (15)

$(H_2O)_3Fe(CO_3) + HSO_5^-/S_2O_8^{2-} \rightarrow Fe^{III}_{aq} + CO_3^{\cdot -} + SO_4^{2-}/2SO_4^{2-}$ (small slope) (16)

The overall rate law for this reaction (rds (16)) is as follows:

$r = k_{16} [(H_2O)_3Fe(CO_3)] [HSO_5^-/S_2O_8^{2-}]$ (16')

From (15),

$[(H_2O)_3Fe(CO_3)] = K_{15}[Fe(H_2O)_6^{2+}] [HCO_3^-]$

Eq. (16') may be rewritten as,

**rate = $\mathbf{k_{16}K_{15}[Fe(H_2O)_6^{2+}] [HCO_3^-] [HSO_5^-/S_2O_8^{2-}]}$**

***Large slope***

$Fe(H_2O)_6^{2+} + 2HCO_3^- \rightleftharpoons (H_2O)_2Fe(CO_3)_2^{2-} + 2H_3O^+ + 2H_2O$ (17)

$(H_2O)_2Fe(CO_3)_2^{2-} + HSO_5^-/S_2O_8^{2-} \rightarrow Fe^{III}(CO_3)_{aq} + CO_3^{\cdot -} + SO_4^{2-}/2SO_4^{2-}$

(large slope) (18)

Overall rate law (rds (18)) will be

$r = k_{18}[(H_2O)_2Fe(CO_3)_2^{2-}] [HSO_5^-/S_2O_8^{2-}]$ (18')

From (17),

$[(H_2O)_2Fe(CO_3)_2^{2-}] = k_{17} [Fe(H_2O)_6^{2+}] [HCO_3^-]^2 / k_{-17}$

Eq. (18') may be rewritten as,

**rate = $\mathbf{k_{18}K_{17}[Fe(H_2O)_6^{2+}] [HCO_3^-]^2 [HSO_5^-/S_2O_8^{2-}]}$**

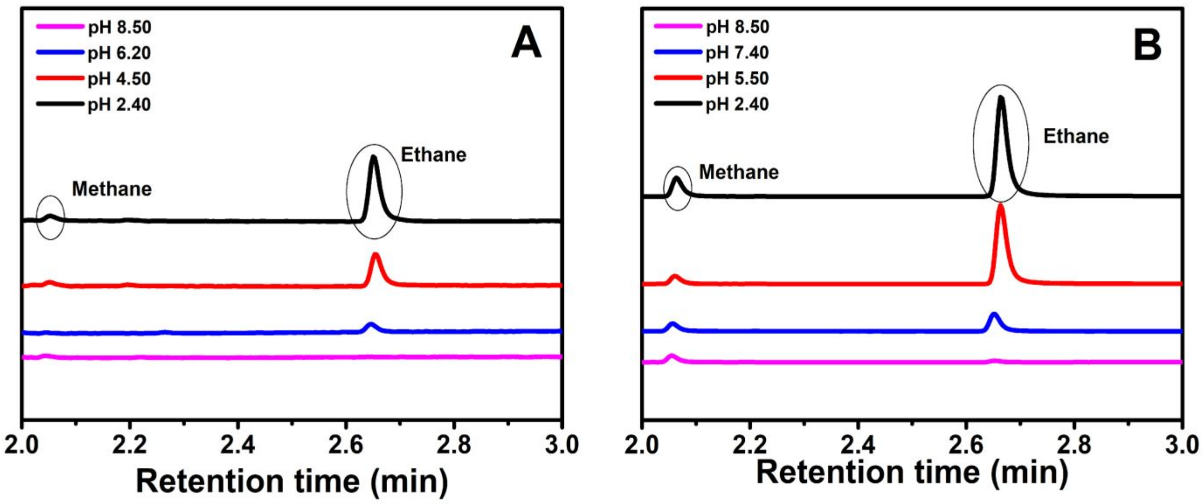


**Figure S14**. GC determination of methane and ethane with FID detector at four different pH in the absence of $HCO_3^-$ after the Fenton reaction of (**A**) $Fe^{2+}$ and $HSO_5^-$ and (**B**) $Fe^{2+}$ and $S_2O_8^{2-}$ in presence of excess $Fe^{II}_{aq}$ and $(CH_3)_2SO$. $[Fe^{II}] = 0.50$ mM, $[HSO_5^-/S_2O_8^{2-}] = 0.10$ mM, $[(CH_3)_2SO] = 25.0$ mM.

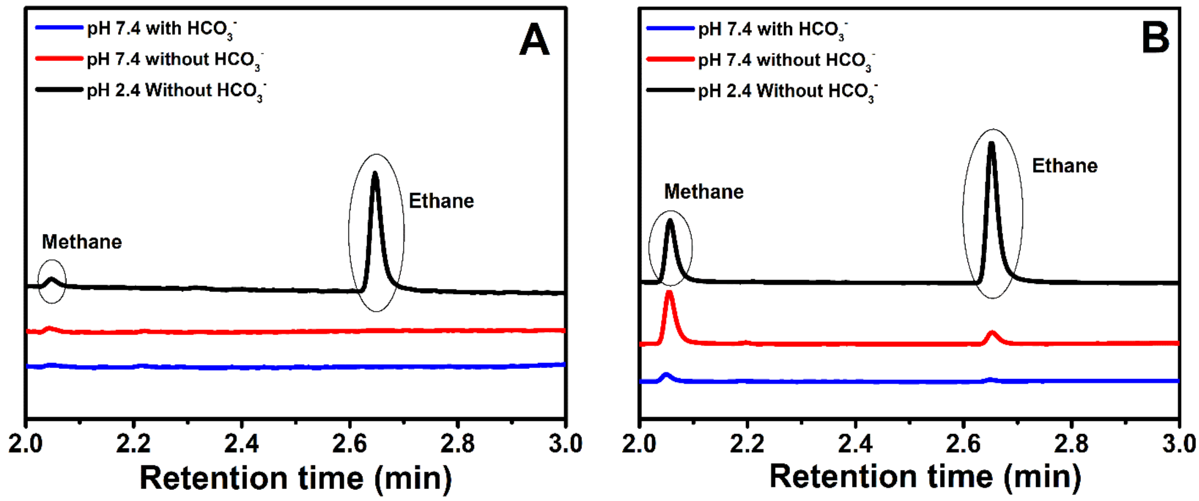


**Figure S15**. GC determination of methane and ethane with FID detector at pH 2.2 and at neutral pH in the absence and presence of $HCO_3^-$ after the Fenton reaction of (**A**) $Fe^{2+}$ and $HSO_5^-$ and (**B**) $Fe^{2+}$ and $S_2O_8^{2-}$ in presence of excess $Fe^{II}_{aq}$ and $(CH_3)_2SO$. $[Fe^{II}] = 0.50$ mM, $[HSO_5^-/S_2O_8^{2-}] = 0.10$ mM, $[(CH_3)_2SO] = 25.0$ mM, $[HCO_3^-] = 3.0$ mM.

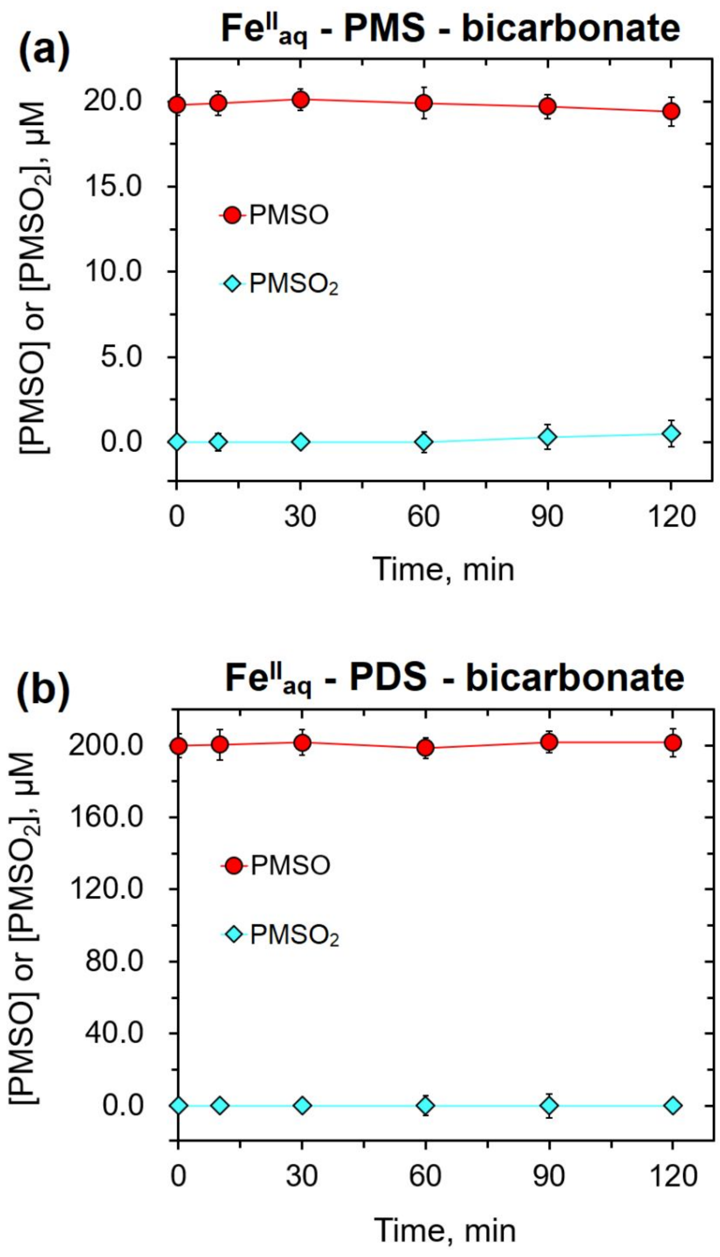


**Figure S16.** The changes in concentrations of PMSO and $PMSO_2$ in the **(a)** $Fe^{II}_{aq}$ - PMS and **(b)** $Fe^{II}_{aq}$ - PDS systems in presence of high bicarbonate concentrations (Initial pH = 7.0. The PMS system: $[PMS]_0$ = 0.04 mM; $[Fe^{II}_{aq}]_0$ = 0.2 mM; $[bicarbonate]_0$ = 20.0 mM; $[PMSO]_0$ = 20.0 μM. The PDS system: $[PMS]_0$ = 1.0 mM; $[Fe^{II}_{aq}]_0$ = 2.0 mM; $[bicarbonate]_0$ = 200.0 mM; $[PMSO]_0$ = 200.0 μM)).

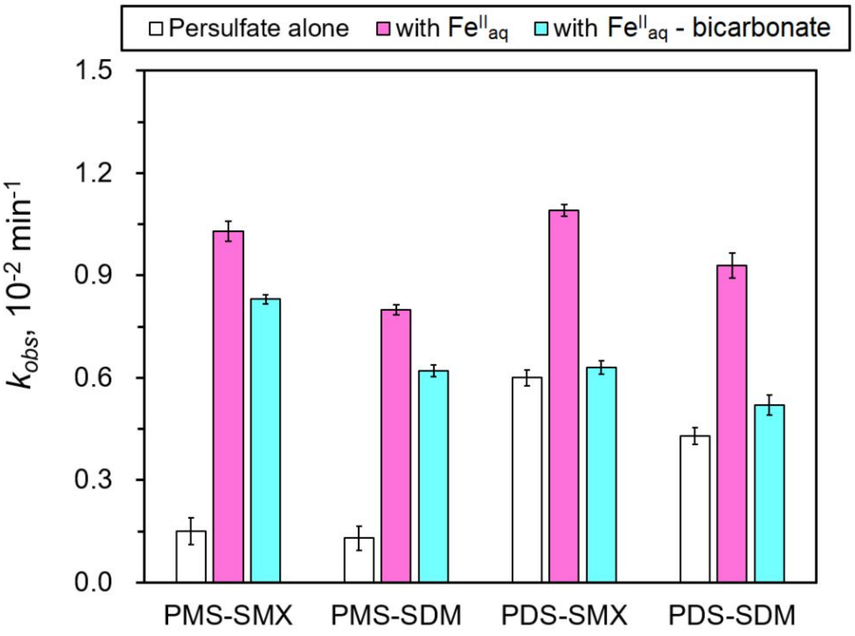


**Figure S17.** The pseudo-first-order rate constants of the degradation of SMX and SDM by different persulfate systems ($[SMX]_0$ = $[SDM]_0$ = 5.0 μM; initial pH = 7.0. The PMS system: $[PMS]_0$ = 0.04 mM; $[Fe^{II}_{aq}]_0$ = 0.2 mM; $[bicarbonate]_0$ = 0.5 mM. The PDS system: $[PDS]_0$ = 1.0 mM; $[Fe^{II}_{aq}]_0$ = 2.0 mM; $[bicarbonate]_0$ = 5.0 mM.).
